# From marine neglected substrata new fungal taxa of potential biotechnological interest: the case of *Pelagia noctiluca*

**DOI:** 10.3389/fmicb.2024.1473269

**Published:** 2024-10-11

**Authors:** Marcella Pasqualetti, Martina Braconcini, Paolo Barghini, Susanna Gorrasi, Domenico Schillaci, Donatella Ferraro, Gerardo Della Sala, Simona De Marino, Massimiliano Fenice

**Affiliations:** ^1^Department of Biological and Ecological Sciences, University of Tuscia, Viterbo, Italy; ^2^Laboratory of Ecology of Marine Fungi (CoNISMa), University of Tuscia, Viterbo, Italy; ^3^Department of Biological, Chemical and Pharmaceutical Sciences and Technologies (STEBICEF), University of Palermo, Palermo, Italy; ^4^Microbiology Section, Department of Health Promotion, Mother and Child Care, Internal Medicine and Medical Specialties “G. D’Alessandro”, University of Palermo, Palermo, Italy; ^5^Department of Eco-Sustainable Marine Biotechnology, Stazione Zoologica Anton Dohrn, Naples, Italy; ^6^Department of Pharmacy, University of Naples “Federico II”, Naples, Italy; ^7^Laboratory of Applied Marine Microbiology (CoNISMa), University of Tuscia, Viterbo, Italy

**Keywords:** marine fungi, epizoic mycobiota, *Pelagia noctiluca*, antimicrobial activity, Mediterranean Sea, NRPS and PKS genes

## Abstract

**Introduction:**

The marine environment is extremely complex and exerts strong evolutionary pressure often leading to the appearance of microbial strains with new metabolic competencies. Microorganisms in marine ecosystems are still largely unknown and should be explored and conserved for biodiversity preservation, possible ecosystem restoring, and other applications. Biodiversity conservation should become a basic ecological strategy of particular significance in relation to global change. In this context, the present research aimed at exploring the culturable mycobiota associated with the jellyfish *Pelagia noctiluca,* never studied before. In addition, the isolated strains were tested for potential application (antimicrobial activity and presence of genes related to the production of secondary metabolites).

**Methods:**

Five jellyfishes were collected in the coastal area of Giglio Island and processed to isolate epizoic fungi. The strains were identified using a polyphasic approach (morphological, physiological, and molecular) and their salt preference was also investigated. The antifungal and antibacterial activity were tested for each strain with agar plug diffusion test. The presence of some key genes related to the main pathways for the production of secondary metabolites in fungi, polyketide synthases (PKSs), and non-ribosomal peptide synthase (NRPSs), was also assessed.

**Results:**

A total of 164 isolates were obtained; after the dereplication, 40 morphotypes, and 23 species were identified. The phylogenetic analyses suggested the presence of new taxa belonging to Pleosporales: two new genera and species, and a new species of *Tamaricicola*. The detected mycobiota showed a relatively high diversity, if compared to other epizoic fungal communities. All isolated strains were marine fungi as confirmed by their salt preference and marked euryhalinism. The genes related to the two main pathways for the production of secondary metabolites in fungi, PKSs and NRPSs, were identified in four and nine strains, respectively. The antimicrobial activity was revealed in 70% of the strains, including the new taxa. The abundance of bioactive strains may be related to the potential involvement of epizoic fungi in host defense strategies. Moreover, these strains could show a high potential for further biotechnological applications particularly in the case of new taxa. All strains are maintained in culture collections.

## 1 Introduction

The marine environment is extremely complex showing several physio-chemical factors (salinity, low water activity, high concentrations of ions, etc.) exerting strong evolutionary pressure, often leading to the appearance of strains with new metabolic competencies (Raghukumar, 2008; Damare et al., 2011; Burgaud et al., 2022). Biodiversity in marine ecosystems is still largely unknown, in particular concerning the structure, composition, and functionality of the microbial communities. This is partly due to the technical hurdles of collecting accurate samples, primarily in open areas, across various marine ecosystems (Templado et al., 2010). In addition, there is still relatively little microbiological research effort, mainly considering the great heterogeneity of habitats, substrata, and ecological niches (Costello and Chaudhary, 2017). The effects of marine habitat constraints are particularly noticeable in organisms like fungi that, to survive, must adopt specific strategies to exert environmental “biochemical” control (bioactive compounds) to compete with other organisms (Jones et al., 2022; Pasqualetti et al., 2022). Over the past few decades, a number of novel and/or uncommon enzymes and metabolites from marine fungi have been discovered (Parte et al., 2017; Birolli et al., 2019; Carroll et al., 2019; Pasqualetti et al., 2019; Giovannini et al., 2019; Botta et al., 2020; Pasqualetti et al., 2022).

The search for bioactive molecules has been carried out by traditional culture-dependent approaches for many years. The advent of molecular methods allowed for improvement and sped out this process with a functional gene-based strategy of molecular screening overcoming some limitations of the traditional culture-dependent approach. In addition, the study of the targeted genes consents to find silent or cryptic gene clusters that potentially encode several bioactive metabolites (Bentley et al., 2002). The two main pathways for the production of secondary metabolites involve the non-ribosomal peptide synthase (NRPS) and polyketide synthase (PKS). It has been reported that fungi with PKS or NRPS genes showed valuable secondary bioactive metabolites (Cox and Simpson, 2009; Vassaux et al., 2019).

Furthermore, applied mycological investigations often do not provide taxonomic and ecological information regarding the studied organisms (Pang et al., 2018; Le et al., 2019; Shaker et al., 2021; Ren et al., 2022; Ye et al., 2022). In particular, the fungi are frequently described as “marine-derived” without providing any information to state the strain’s marine habitus (Pasqualetti et al., 2020). However, these inquiries are crucial for understanding the fungal biodiversity and the exact role of marine fungi in the sea ecosystems. Thus, despite the recent advancements in marine mycology, the scientific community is just beginning to understand the importance of fungi in the marine system and glimpse the complexity of their ecosystem services (Amend et al., 2019; Grossart et al., 2019). Despite the recent improvements in marine mycology, several taxonomical and ecological issues still need to be addressed. Among them, the main topics are: a deeper knowledge of fungal diversity and a formal description of new taxa, a broader characterization of the mycobiota associated with poorly studied or neglected substrata, and a wider comprehension of the ecological role of marine biotic fungi.

It is crucial to underline that the unknown biodiversity represents an important gap in understanding ecosystem functioning and a huge potential resource that could not be lost. One of the main instruments to address these topics is to promote the collection activities and best conservation practices, mainly regarding new taxa, and new marine strains of common terrestrial species. Biodiversity conservation, *in situ* and *ex situ,* is one of the main actions to contrast global change (Hagerman et al., 2010).

Recently, studies on the mycobiota associated with various substrata evidenced that marine fungi, like the terrestrial ones, exhibit some degree of specialization. This is more evident in epibiotic species since some of them are ubiquitous, others are specialized and strongly associated with a specific substratum, while others have intermediate patterns (Pasqualetti et al., 2014; Bovio et al., 2018; Nguyen and Thomas, 2018; Pasqualetti et al., 2020). Gao et al. (2008) showed great differences in the mycobiota composition associated with two coexisting Hawaiian marine sponges. Similar species-specificity has been observed in fungi associated with seagrass and seaweeds (Wainwright et al., 2017; Pasqualetti et al., 2020), in mycorrhizae-like associations in seagrass roots (Borovec and Vohnik, 2018), and in scleractinian corals (Williams et al., 2014). Although the nature of these interactions remains unclear, bioactive molecules may play significant roles in fungal interactions with marine hosts. Zhou et al. (2011) suggested that the presence of PKS or NRPS genes in epi-/endo-zoic fungi could have a potential role in the chemical host defense (Raghukumar and Ravindran, 2012).

Fungi associated with marine animals have received special attention since they are frequently found to be a valuable biotechnological resource, mainly due to the production of bioactive molecules (Marchese et al., 2020). Only a limited number of studies considered jellyfish mycobiota, and in general, the investigations did not focus on communities but only on the production of secondary metabolites by single strains (Yue et al., 2022; Li et al., 2023). For instance, some of the strains isolated from *Nemopilema nomurai* produced several new bioactive compounds and a new molecule of great interest (epicoccamide) was isolated from an epi-biont of *Aurelia aurita* (Wright et al., 2003; La Kim et al., 2012a,b). Although jellyfish-associated marine fungi have shown to be an unexploited source of novel molecules of biotechnological importance, the role of these metabolites in the interactions with their host remains almost unknown. Therefore, much more effort should be made to study the fungal community associated with different species of jellyfish and to understand the nature of these interactions.

*Pelagia noctiluca* (Cnidaria: Scyphozoa), known as the “mauve stinger,” is considered one of the most common jellyfish in the Mediterranean Sea (Mariottini et al., 2008; Canepa et al., 2014). *Pelagia noctiluca* is a pelagic organism generally pink-, mauve-, or light brown-colored, with a phosphorescent bell characterized by a thick jelly hemispherical umbrella, up to 12 cm in diameter with an exoumbrella with several nematocyst warts. It presents eight adradial marginal tentacles alternated with eight marginal rhopalia, four inter-radial gonads, and oral arms (Tibballs, 2006; Durgham et al., 2016). *Pelagia noctiluca* is a predator that feeds on several zooplankters, including eggs and larvae of nektonic and benthic organisms. This jellyfish performs daily vertical migrations, staying at the surface at night and sinking in deeper water during the day. This vertical distribution pattern coincides with the migration of zooplankton, which represents its main prey (Tilves et al., 2016).

To the best of our knowledge, there are no studies regarding the *P. noctiluca* mycobiota.

This work was aimed at studying the culturable assemblages of fungi related to *P. noctiluca* collected in a *Posidonia* meadow located at the Giglio Island (Tyrrhenian Sea, Italy). Marine fungi were isolated, identified, and taxonomically characterized with a polyphasic approach including morphological, physiological, molecular, and phylogenetic analysis. In addition, for a better comprehension of the *P. noctiluca* mycobiota ecological role and its possible biotechnological applications, presence of NRPS and PKS genes and preliminary antimicrobial activity was studied.

## 2 Materials and methods

### 2.1 Strain isolation

Samples of the jellyfish *Pelagia noctiluca* were collected from the “Cala Cupa” cove (4222008.0300 N, 1055004.0900E), Giglio Island (Tuscan Archipelago, North Tyrrhenian Sea) at 10 m depth near a previously studied meadow in May 2019. After capture by scuba divers, five entire living jellyfishes were placed separately in sterile containers and then stored in a refrigerator (icebox), and brought to the laboratory. The five animals were asymptomatic and well-developed, with equivalent size (umbrella range 9–11 cm in diameter). The samples were prepared for the isolation of fungi as follows. The samples were washed (five time) with sterile seawater to remove sediments, debris, and transient microorganisms (i.e., propagules not strictly associated with jellyfish) and gently blotted with sterile filter paper to remove water (Yue et al., 2015). To evaluate the effectiveness of this procedure in removing all the transient propagules, all washing solutions were plated on Potato Dextrose Agar Sea water (PDAs: PDA 39 g—Sigma-Aldrich, St. Louis, MO, United States—dissolved in 1 L of filtered seawater) and incubated at 20°C; to assess possible propagule growth (no growth was observed plating the last rinsing solution). Samples of the umbrella (U) and oral arms (OA) were excised manually from each animal and separately placed into sterile glass Petri dishes (80 mm × 15 mm). The inner tissues (IT) of the animals, including gonads, were also removed and placed in sterile Falcon tubes (50 mL).

In order to optimize the isolation procedures for the umbrellas and the oral arms, some preliminary tests were carried out to compare the homogenization and the direct plating methods. The results demonstrated that the isolation yield using the homogenization method was definitely lower than that achieved by the direct plating. Moreover, all the fungal species isolated by the homogenization method were also found by direct plating. Accordingly, the isolation was carried out using two different techniques for the different districts of the animals: “direct plating” for OA and U, and “homogenization and plating” for IT, within 24 h from sampling.

Oral arms and U samples were cut into small pieces (10 pieces of *ca*. 5 mm^2^ for each substratum and sample) and directly placed on Petri dishes (five pieces for each plate 9 cm Ø) containing PDAs. Twenty Petri dishes were set up for OA and U.

For IT, 5 g (1 g from each animal) of tissues was added to 10 mL of sterile seawater and homogenized (ULTRA-TURRAX®-IKA, Staufen, Germany). The homogenate and 1:10 and 1:100 dilutions, in sterile sea water, were used to inoculate the plates: 0.5 mL of each dilution was spread in five Petri plates (9 cm Ø) containing PDAs. Five plates for each substratum and dilution were performed.

To prevent bacterial growth all media were supplemented with an antibiotic mix of 0.2 g/L streptomycin sulfate and 0.007 g/L penicillin G (Sigma-Aldrich, St. Louis, MO, United States). All procedures were carried out under sterile conditions.

A total of 50 plates were incubated at 20°C in the dark for up to 6 weeks and regularly monitored. All developed colonies were collected, transferred on appropriate media, and isolated in pure cultures. Rejection of duplicates (dereplication) was carried out by preliminary morphological, taxonomical and physiological analyses on different cultural media: PDAs, Corn Meal Agar Sea water (CMAs: 17 g CMA—Sigma-Aldrich dissolved in 1 L of filtered sea water), Malt Extract Agar Sea water (MEAs: 50 g MEA—Sigma-Aldrich dissolved in 1 L of filtered seawater), and Czapeck Sea water (CZs: 49 g CZ—Sigma-Aldrich, dissolved in 1 L of filtered seawater). However, every strain showing any morphological differences at macroscopic (colony) or microscopic (reproductive structures: asexual conidia, conidiphores, conidioma, or sexual structures) level were maintained as different morphotypes. The same dereplication approach was used considering physiological features revealed by the growth on different substrata (rate of growth, texture of the colonies, exudate, and pigment production).

All different morphotypes were cryo-conserved (Cryoinstant Mixed, VWR, Leuven, Belgium) and maintained in the culture collection of the “Laboratory of Ecology of Marine Fungi,” (CoNISMa) Department of Ecological and Biological Sciences (DEB), University of Tuscia, Viterbo, Italy. The strains of new taxa were also deposited at the international public institution, *Mycotheca Universitatis Taurinensis* (MUT).

### 2.2 Strain identification

A polyphasic approach that included morpho-physiological, molecular, and phylogenetic analyses was used to identify fungal strains.

#### 2.2.1 Morpho-physiological identification

The strains were first identified at the genus level, and then at the species level when possible, using morpho-physiological identification based on macroscopic, microscopic, and physiological features (Ellis, 1971, 1976; Kohlmeyer and Kohlmeyer, 1979; Pitt, 1979; Sutton, 1980; Hyde et al., 2000; Klich, 2002; Domsch et al., 2007; Jones et al., 2015).

#### 2.2.2 Molecular identification

Genomic DNA was extracted using the ZR Fungal/Bacterial DNA MiniPrep Kit (Zymo Research, Irvine, CA, United States) according to the manufacturer’s instructions. The quantity of DNA was spectrophotometrically quantified (Qubit, Thermo Fisher Scientific, Waltham, MA, United States), and DNA samples were stored at −20°C. For each fungal strain, the ITS rDNA (ITS1-5.8S-ITS2) was amplified using the universal primers ITS5 and ITS4 (White et al., 1990). Based on the preliminary taxonomic assignment, other specific primers were selected as reported in Table 1. Amplifications were run in a 2720 Thermal Cycler (Applied Biosystem, Waltham, MA, United States) (Table 1).

**Table 1:** PCR amplification protocols used for the different markers.

Marker/Primers	Strains	Thermocycler conditions	References
nrITS	All	94°C × 2 min, (94°C × 40 s, 55°C: 30 s, 72°C: 45 s) × 35 cycles; 72°C: 10 min	White et al. (1990)
ITS5/ITS4			
nrLSU	PN6, PN9, PN27, PN33	95°C: 10 min, (95°C: 60 s, 50°C: 30 s, 72°C: 90 s) × 40 cycles; 72°C: 10 min	Vilgalys and Hester (1990)
LR0R/LR7			
nrSSU	PN1, PN6, PN9, PN27, PN33	95°C: 10 min, (95°C: 60 s, 50°C: 30 s, 72°C: 90 s) × 40 cycles; 72°C: 10 min	White et al. (1990)
NS1/NS4			
*act*	PN3, PN16, PN17, PN29, PN43, PN44	95°C: 8 min (95°C:15 s, 65°C: 20 s, 72°C: 60 s) × 35 cycles; 72°C: 5 min	Carbone and Kohn (1999)
ACT-512F/ACT-783R			
*β-tub*	PN1, PN9, PN15, PN20, PN22, PN25, PN31, PN33, PN35	94°C: 5 min, (94°C: 35 s, 56°C: 55 s, 72°C: 60 s) × 35 cycles; 72°C: 10 min	O’Donnell and Cigelnik (1997)
T1/T22			
*CaM*	PN20	94°C: 2 min, (94°C: 20 s, 55°C: 30 s, 72°C: 45 s) × 35 cycles; 72°C: 10 min	Peterson (2004)
CF1/CF4			
*tef-1α*	PN1, PN3, PN6, PN7, PN9, PN27, PN31, PN33, PN38, PN40	95°C: 5 min, (95°C: 30 s, 54°C: 50 s, 72°C: 60 s) × 40 cycles; 72°C: 10 min	Rehner and Buckley (2005)
EF-983F/EF-2218R			
*tef-1α*	PN1, PN3, PN6, PN7, PN9, PN27, PN31, PN33, PN38, PN40	95°C: 10 min, (95°C: 30 s, 55°C: 30 s, 72°C: 60 s) × 40 cycles; 72°C: 10 min	Rehner and Buckley (2005)
EF-728 M/EF-2			
*rpb-2*	PN1, PN6, PN7, PN9, PN27, PN31, PN33, PN38, PN40	94°C: 5 min, (94°C: 45 s, 60°C: 45 s, 72°C: 120 s) × 5 cycles; (94°C: 45 s, 58°C: 45 s, 72°C: 120 s) × 5 cycles; (94°C: 45 s, 54°C: 45 s, 72°C: 120 s) × 30 cycles; 72°C: 8 min	Liu et al. (1999)
fRPB2-5F/fRPB2-7CR			
G3PDH	PN7, PN40	94°C: 5 min, (94°C: 30 s, 64°C: 30 s, 72°C: 90 s) × 35 cycles, 72°C: 10 min	Staats et al. (2005)
G3PDHF/G3PDHR			
HSP60	PN7, PN40	94°C: 5 min, (94°C: 30 s, 55°C: 30 s, 72°C: 90 s) × 35 cycles, 72°C: 10 min	Staats et al. (2005)
HSP60F/HSP60R			
PKS	All	94°C × 3 min, (94°C × 60 s, 55°C: 60 s, 72°C: 180 s) × 34 cycles; 72°C: 10 min	Bingle et al. (1999)
LC1/LC2c			
LC3/LC5c			
NRPS	All	94°C × 3 min, (94°C × 60 s, 55°C: 30 s, 72°C: 180 s) × 40 cycles; 72°C: 10 min	Slightom et al. (2009)
AUG003/AUG007			
AUG005/AUG007			

nrITS, Internal transcribed spacer; nrLSU, Large ribosomal SubUnit; nrSSU, Small ribosomal SubUnit; *act*, actin; *β-tub*, β-tubulin; *CaM*, Calmodulin; *tef-1α*, Trans elongation factor; *rpb-2*, RNA polymerase II subunit; G3PDH, Glyceraldehyde-3-phosphate dehydrogenase; HSP60, Heath-Shock Protein 60; PKS, Polyketide synthases; NRPS, Non-ribosomal peptide synthetase.

PCRs were performed in a volume of 25 μL mixture containing: 0.5 μL of each primer (0.2 μM), 2.5 μL of MgCl_2_ (25 mM), 1.5 μL of 5 x buffer, 0.5 μL of dNTPs (0.2 mM), 0.2 μL of Go-Taq Polymerase (Promega, Madison, WI, United States), and 2 μL of genomic DNA; the final volume (25 μL) was reached adding ultrapure water. The PCR products were purified (E.Z.N.A. Cycle Pure kit Omega Bio-tek, Norcross, GA, United States) and sent to Eurofins Genomics (Ebersberg, Germany) for sequencing. Sequences obtained were checked and trimmed with Chromas Lite 2.1 program and then compared with those deposited in GenBank NCBI (National Center for Biotechnology Information, Bethesda, MD, United States). Newly generated sequences were deposited in GenBank, the accession numbers are reported in Table 2.

**Table 2:** Taxonomical attribution of the isolates from *Pelagia noctiluca*; strains and isolation districts, physiological characters (salinity tolerance, in brackets salinity optimum) and GenBank accession number of the obtained sequences.

Taxon	Code	Isolation districts			Salinity (opt.) ‰	Molecular markers and GenBank accession numbers						
		OA	IT	U		nrITS	*act*	*β-tub*	nrLSU	nrSSU	*tef*1-α	*rpb*-2
Dothideomycetes												
*Cladosporium aggregatocicatricatum*	**PN29**		x		0 > 120 (35)	MZ221935	MZ305182					
*C. allicinum*	**PN43**			x	0 > 120 (35)	MZ221774	MZ305181					
	**PN10**	x				OP793782						
*C. delicatulum*	**PN16**		x		0 > 120 (35)	MZ221944	MZ305184					
	**PN26**			x		OP793881						
	**PN42**			x		OP793885						
	**PN08**			x		OP793794						
	**PN12**	x	x			OP793841						
	**PN19**		x			OP793882						
	**PN11**	x				OP793798						
*C. halotolerans*	**PN17**		x		0 > 120 (35)	MZ221974	MZ305183					
*C. myrtacearum*	**PN03**		x		0 > 120 (35)	MZ221760	MZ305185				ON979657	
*C. westerdijkiae*	**PN44**			x	0 > 120 (35)	MZ222249	MZ291568					
*Keissleriella* sp.	**PN06**		x		0 > 120 (35)	ON807306			ON838727	ON839999	ON952517	ON866518
											ON979660	
*Neopyrenochaeta acicola*	**PN31**		x		0–120 (0)	ON806621		ON866492			ON952518	ON866517
											ON979661	
*Neovaginatispora fuckelii*	**PN27**			x	0–120 (35)	ON807307			ON838938	ON840004	ON952519	ON887329
											ON979662	
Phaeosphaeriaceae sp.1	**PN09**			x	0–120 (0)	ON807308		ON866494	ON838726	ON840001	ON952521	ON887327
											ON979664	
Phaeosphaeriaceae sp.2	**PN33**		x		0–120 (0)	ON807308		ON866495	ON838942	ON840006	ON952520	ON887330
*Roussoella intermedia*	**PN01**		x		0–120 (0)	ON805845		ON866493		ON839998	ON952523	ON866516
											ON979665	
*Tamaricicola* sp. IG 108	**PN38**		x		0 > 120 (0/35)	ON807355		MZ322901			ON952522	ON887328
											ON979666	
	**PN39**		x			OP794025						
	**PN23**		x			OP793911						
	**PN28**		x			OP793912						
	**PN32**		x			OP793995						
Eurotiomycetes												
*Penicillium antarcticum*	**PN35**		x		0 > 120 (35)	MZ254898		ON866496				
	**PN14**		x			OP793893						
*P. bialowiezense*	**PN22**		x		0 > 120 (70)	MZ254911		ON866500				
*P. brevicompactum*	**PN25**		x		0 > 120 (35/100)	MZ254900		ON866497				
	**PN37**		x			OP793895						
	**PN13**	x				OP793896						
*P. fundyense*	PN20*		x		0 > 120 (35)	MZ254909		ON866499				
	**PN24**		x			OP793891						
*P. polonicum*	**PN15**		x		0 > 120 (35)	MZ254904		ON866498				
Leotiomycetes												
*Botrytis caroliniana*	PN40*	x			0–120 (0)	ON807238					ON979658	ON887326
	**PN41**			x		-						
*B. cinerea*	**PN02**		x		0–100 (0)	OP793783						
	PN07*			x		ON807239					ON979659	ON887325
Sordariomycetes												
*Chaetomium elatum*	**PN36**		x		0 > 120 (35)	MZ222288		MZ322901				
Tremellomycetes												
*Papiliotrema* sp.	**PN21**		x		0 > 120 (0)	MZ254988						
Microbotryomycetes												
*Sporobolomyces roseus*	**PN04**		x		nd	MZ221766						

OA, Oral arms; IT, Inner tissue; U, Umbrella; *Additional markers were amplified for some strains. The CaM gene was amplified for strain PN20 (ON952524); G3PDH was amplified for strains PN7 (OP019700) and PN40 (OP019701); HSP60 gene was amplified for strains PN7 (OP019698) and PN40 (OP019699); nd, Not detected.

Taxonomic assignment was based on similarity (NCBI-BLASTn algorithm) to reference sequences available at GenBank; similarity values higher than 98% (*e*-value > *e*_100) were considered reliable, attributions were confirmed by morphological and phylogenetic analyses.

Phylogenetic analysis was carried out on the nrITS region for all morphotypes; additional analyses, based on the *actin* (*act*) for Capnodiales, and *β-tubulin* (*β-tub*) for Eurotiales, were also performed.

Multi-locus phylogenetic analyses were performed, using concatenate datasets, based on nrITS region, nrLSU and nrSSU for Pleosporales and on *rpb*-2, G3PDH, and HSP60 for Leotiomycetes (Staats et al., 2005; Phookamsak et al., 2019). The references of all sequences included in the datasets are reported in Supplementary Tables S1–S5.

Sequences were aligned with the Clustal X 2.1 software (Thompson et al., 1997) using the default parameters. Alignments were checked and edited with BioEdit Alignment Editor 7.2.5 (Hall, 1999) and manually adjusted in MEGA X. For the multi-locus phylogenetic analyses, alignments of different markers were concatenated into a single data matrix with MEGA X. Phylogenetic inference was estimated using Maximum Likelihood (ML) and Bayesian inference (BI) as previously reported by Braconcini et al. (2024). ML analyses (10,000 bootstrap replicates) were run using the IQ-TREE web server, the best-fit evolution model was inferred using the ModelFinder in IQ-TREE; different models were used for each partition in the concatenated matrices (Trifinopoulos et al., 2016; Kalyaanamoorthy et al., 2017). The best-scoring trees were visualized using FigTree v.1.4 (FigTree (2024): Tree Figure Drawing Tool Version 1.4.4., 2024). The BI was performed with MrBayes 3.2.7 under different models for each partition as evaluated by jModelTest 2 (Posada, 2008) using Bayesian Information Criterion (Ronquist et al., 2012). The alignment was run for 1 million generations in two independent runs, each with four Markov Chains Monte Carlo (MCMC) and sampling every 100 iterations. As a “burn-in” measure, the first 25% of generated trees were discarded. MrBayes’ “sumt” function was used to generate a consensus tree, and Bayesian posterior probabilities (BYPP) were calculated.

Sequence alignment and phylogenetic tree were deposited in TreeBASE version 2 (2024) (submission ID: 31592). For each fungal species, the current name according to the “*Index Fungorum*” database (Index Fungorum database and web site, 2024) and recent literature was reported.

### 2.3 Determination of growth at different salinities

The effect of salinity on fungal growth was investigated on PDA modified with different NaCl concentrations: 0, 35, 70, 105, and 120‰. The plates (90 mm Ø) were inoculated with a single agar disk (diameter 3 mm) cut from the actively growing margin of 7-day-old colonies grown on PDAs. Plates were incubated at 25°C and colony diameter was measured daily for 7–30 days (in relation to the different growth rates) to estimate fungal growth. Experiments were carried out in triplicate.

### 2.4 Antimicrobial bioassay

#### 2.4.1 Test microorganisms

All tests were performed on saline media. The halotolerant strains of *Bacillus pumilus* KB66, *Pseudomonas fluorescens* KB6 (Pesciaroli et al., 2012) and *Penicillium griseofulvum* TSF04 were used as test organisms to avoid interferences related to salt presence. The test strains were retrieved from the culture collection of microorganisms of the Laboratory of Ecology of Marine Fungi. Strains had been revitalized and sub-cultured on Luria Bertani Sea water (LBs) broth (25 g LB—Sigma-Aldrich dissolved in 1 L of filtered seawater) and MEAs for the bacterial and fungal strain, respectively.

To produce standardized inoculum, bacteria were grown in LBs at 30°C in an orbital shaker at 150 rpm. After overnight incubation, the bacteria were diluted by the addition of fresh media to an optical density (OD) of 0.8 at 680 nm. *Penicillium griseofulvum* was grown on MEAs for 7 days at 25°C. Conidia suspensions were prepared in sterile filtered seawater supplemented with 0.01% of Tween 80 and diluted to a final inoculum ranging from 0.5 × 10^5^ to 1.0 × 10^5^ cells/mL. Test plates were inoculated by spreading 100 μL of bacteria culture and 300 μL of spore suspension.

#### 2.4.2 Agar plug diffusion method

The screening for antifungal and antibacterial activity was carried out with the agar plug diffusion method. Fungal strains were cultured on different cultural media (PDA, PDAs, MEA, MEAs, CYA, and CYAs; CYA medium is CZ supplemented with 5 g/L of Yeast Extract) for 90 days at 25°C. The fungal strains were grown for a relatively long period to enhance the production of secondary metabolites as evidenced by some preliminary tests. Agar plugs (3 cm^2^) were aseptically cut at the front of the colony and deposited on the agar surface of plates previously inoculated with the test organisms. Plugs of different cultural media were used as negative control. After incubation (24 h, 30°C for bacteria, and 48–72 h, 25°C for fungi) the antimicrobial activity was estimated by measuring the inhibitions halos.

### 2.5 PKS and NRPS genes screening

Seven PCR primers, designed for the highly conserved sequences of *β*-ketoacyl synthase (PKS) domains and the most conserved A domain of the NRPS gene, were used. PCR were performed as previously reported in section 2.2.2. PCR conditions and primers are reported in Table 1.

### 2.6 Statistical analysis

Principal Component Analysis (PCA) on salt growth preferences were made on the normalized colony diameters on the maximum growth (CANOCO Engine Version 5.2 2017; Šmilauer and Lepš, 2014).

## 3 Results and discussion

### 3.1 Strain isolation and identification

**Figure 1:**
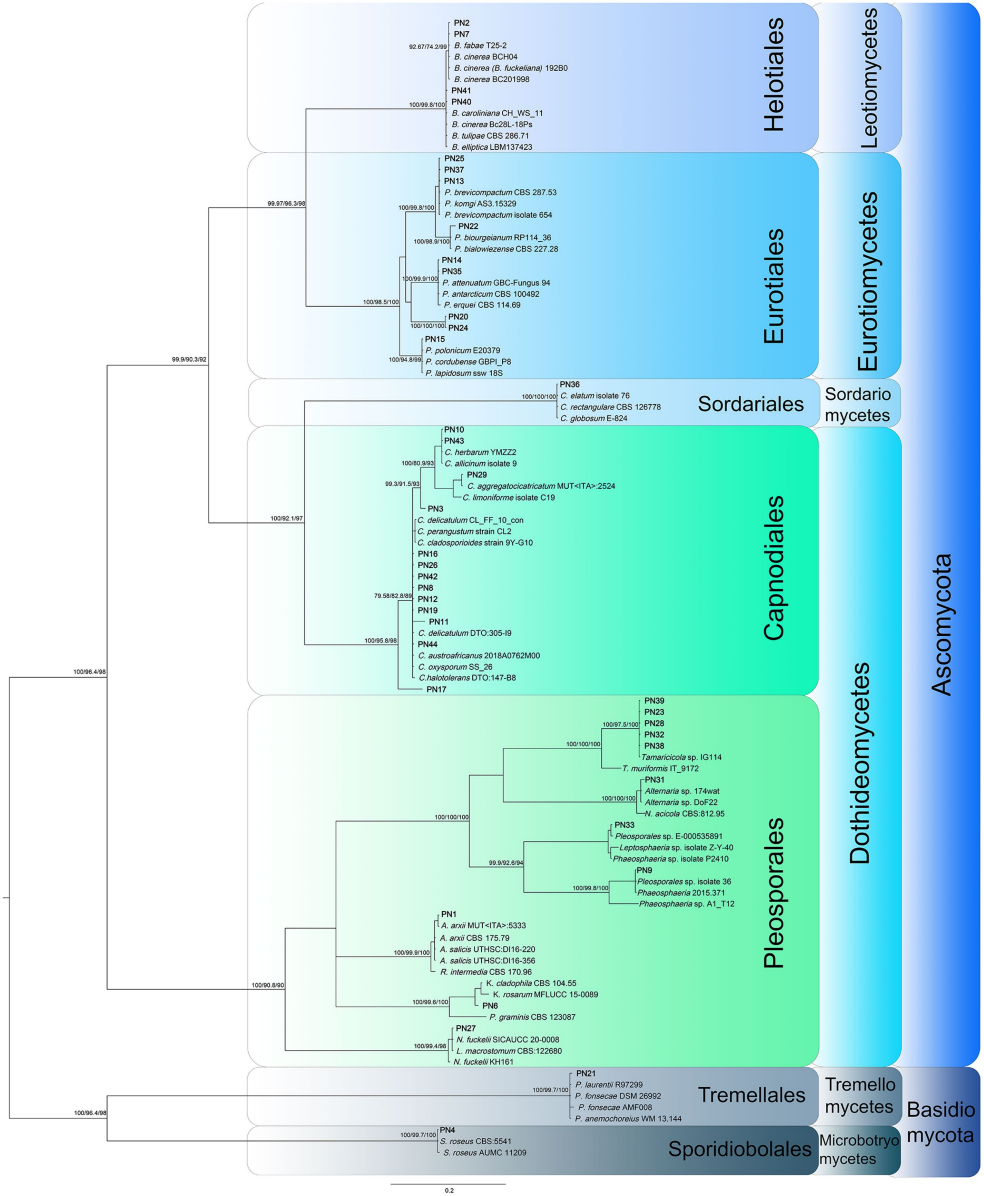
Phylogenetic inference based on ITS1, 5.8S rDNA, and ITS2 partitioned dataset was inferred using the Bayesian method. Branch numbers indicate BYPP values, and sH-aLRT and BP values from RA-ML analysis. The Bayesian Inference was performed under model as evaluated by jModelTest 2 using Bayesian Information Criterion (partition 1: TPM1uf + G, G = 4.445; partition 2: HKY + G, G = 0.140; partition 3: TrNef+G, G = 0.813).

One hundred and sixty-four fungal isolates were obtained from samples of *P. noctiluca*. The fungal assemblages associated to umbrella and oral arms of each jellyfish were quite homogeneous, therefore they were merged to describe the *P. noctiluca* mycobiota. Considering the animals’ district, the inner tissues hosted the highest number of fungal colonizers (89 isolates), followed by umbrella (48 isolates), and oral arms (27 isolates). According to the morpho-physiological dereplication, 40 morphotypes were obtained. The morphotypes were characterized by molecular analysis using the nrITS region, the universal DNA barcode for fungi (Bergerow et al., 2010; Schoch et al., 2012). The analysis of the sequences (BLASTn) and subsequent phylogenetic analysis (Figure 1) allowed to group the 40 morphotypes in 23 distinct taxa. Nine morphotypes were identified at the species level, 26 at the genus level, and for the remaining five strains, all belonging to the Pleosporales order, no further attribution was possible with nrITS region. Considering these results, additional genes were analyzed to attain the attribution at the species level.

All *Cladosporium* and *Penicillium* isolates were identified at the species level by the actin and β-tubulin genes, respectively (Supplementary Figures S1, S2; Bensch et al., 2012; Visagie et al., 2016), with the only exception of PN20. For this morphotype, the analysis of the *CaM* gene was also assessed for its species attribution to *P*. *fundyense*. The β-tubulin gene was also used to confirm the morphological identification of *Chaetomium elatum* PN36. The two *Botrytis* strains PN7 and PN40 were analyzed by a polyphasic approach including both molecular and morphological analyses. A multi-locus phylogenetic analysis was carried out using the *rpb*-2, HSP60, and G3PDH markers, as reported by Staats et al. (2005) to distinguish the most closely related neighbor species (Supplementary Figure S3). The strain PN7 is included in a well-supported group with three different species: *B. cinerea*, *B. eucalypti*, and *B. pelargonii*. Nevertheless, as already observed by Garfinkel (2021), these species must be considered conspecific, thus the strain PN7 was attributed to *B. cinerea*; this attribution was also confirmed by morphological analysis. Also, PN40 is grouped with three different species *B. fabiopsis*, *B. galanthina*, and *B. caroliniana* that have different morphological characters including presence/absence of sclerotia and conidial dimensions (Li et al., 2012). These features lead to the attribution of PN40 to *B. caroliniana* since it does not produce sclerotia, and conidia dimensions are 10–14 × 6–9 μm.

**Figure 2:**
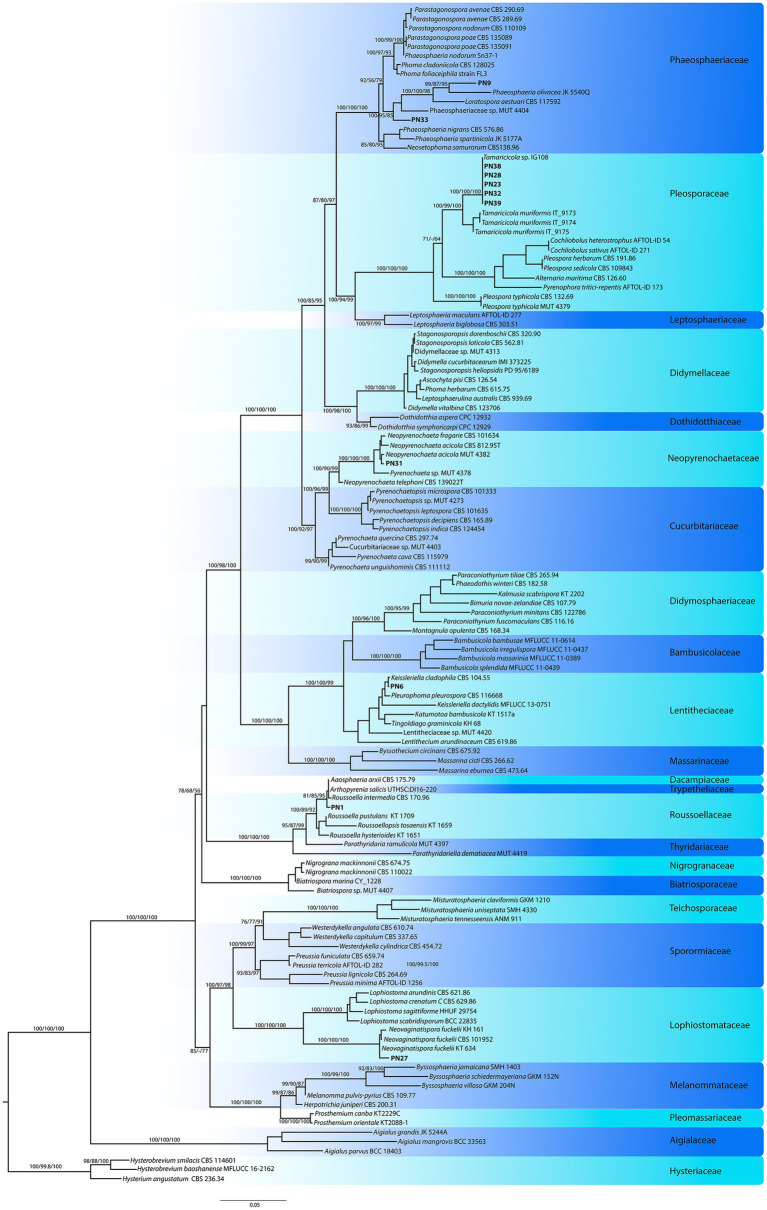
Pleosporales: Phylogenetic inference based on nrLSU, nrSSU, and nrITS (ITS1, 5.8S rDNA, and ITS2) combined dataset was inferred using Maximum Likelihood method (RA-ML), *Hysterobrevium baoshanense* and *H. smilacis* were used as outgroup. Branch numbers indicate BYPP values from Bayesian analysis, and sH-aLRT and BP values from RA-ML analysis. The Maximum Likelihood inference was performed under different models for each partition as evaluated by ModelFinder (IQ-TREE web server): K2P + I + G4 (partition 1), IM2 + F + I + G4 (partition 2), TIM2e + G4 (partition 3); K2P + I (partition 4); TIM2e + I + G4 (partition 5).

A multi-locus phylogenetic analysis based on the nrLSU, nrSSU, and nrITS was used to analyze the 11 morphotypes included in the Pleosporales in order to determine their taxonomic position within this large order (Figure 2). Subsequently, different molecular markers (*β-tub*, *tef*1-*α*, and/or *rpb*-2) were selected and analyzed for each family to reach specific attribution of the strains (Table 2). The pleosporelean morphotypes were included in six families. The strains included in Lentitheciaceae (PN6), Lophiostomataceae (PN27), Neopyrenochaetaceae (PN31), and Roussellaceae (PN1) were attributed to *Keissleriella* sp. (PN6), *Neopyrenochaeta acicola* (PN31), *Neovaginatispora fuckelii* (PN27), and *Roussoella intermedia* (PN1). For PN6, the analyses carried out on nrITS, nrLSU, and nrSSU did not allow to distinguish the morphotype from the related species *K. cladophila*, *K. camporesi*, *K. sparticola*, and *K. rosarum*. The *tef*1-α sequences, efficiently used to attribute some species in the genus *Keissleriella*, are not available for the species strictly related to PN6; in addition PN6 is a *mycelia sterilia* and morphological analysis is useless for species attribution. It is worth to note that some authors reported the inconsistency of some species included in the genus *Keissleriella* and suggested the necessity of a large revision of this genus (Hyde et al., 2020).

The remaining Pleosporales strains included in Pleosporaceae (PN38, PN39, PN23, PN28, and PN32) and Phaeosphaeriaceae (PN9 and PN33) families can be all ascribed to new taxa. The pleosporeacean strains were all included in a strong supported clade with a strain of *Tamaricicola* sp. IG108, previously collected in the same area (Pasqualetti et al., 2020). The clade is strictly related with the monospecific genus *Tamaricicola* and seem to represent a new lineage inside this genus (Figure 2).

The strains PN9 and PN33 represent two new lineages (genera) inside the family Phaeosphaeriaceae (Figure 2). This is also strongly supported by the analyses of genetic distances among PN9 and PN33 and the closest taxa of the family for all tested markers (Supplementary Tables S6, S7).

### 3.2 Growth at different salinities

**Figure 3:**
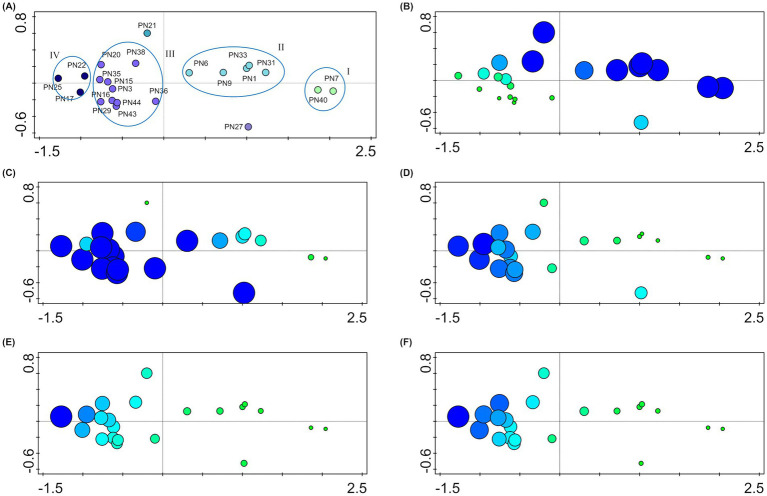
Salinity preferences of the studied strains. **(A)** Multivariate analysis (PCA) carried out on the growth data at different salinities. The plot shows strain distribution related to salinity preferences; four main groups (I, II, III, and IV) are identified from the ellipses. **(B–F)** Attribute plots in ordination space: dimensions and color gradient of the symbols indicate the percentage of growth of each strain at different salinities: 0‰ **(B)**, 35‰ **(C)**, 70‰ **(D)**, 105‰ **(E)**, and 120‰ **(F)**.

According to Pasqualetti et al. (2020) analyses of salinity preferences were carried out to exclude, from the *P. noctiluca* fungal assemblage, the non-marine strains possibly present as propagules derived from terrestrial contamination and unable to growth at the sea salinity. All isolated strains are able to grow at sea salinity and included both halotolerant (36%) and facultative halophilic (64%) fungi (slight—and moderate–facultative halophiles, 56 and 8%, respectively; Gunde-Cimerman and Zalar, 2014; Raghukumar, 2017; Pasqualetti et al., 2020). However, the majority of strains showed their optimal growth at the sea salinity or higher (Table 2). Multivariate analysis carried out on growth data at different salinities, clearly identified four main groups (Figure 3). Strains characterized by optimal growth in the absence of salt or at salinities lower than that of seawater (halotolerant) were included in group I (PN7 and PN40) and group II (PN1, PN6, PN9, PN31, and PN33): Group I presented at sea salinity a growth reduction of 30% and Group II of 20% from the optimum. These strains showed a relatively low halotolerance with an 80–95% growth reduction (from the optimum) at salinities of 120‰. Isolates included in group III (PN16, PN36, PN29, PN43, PN44, PN3, PN35, PN15, PN20, and PN38) showed optimal growth at the sea salinity (35‰) and could be considered as facultative halophiles. Group IV included three strains, PN17, PN22, and PN25 presenting optimal growth at 70‰ (moderate-halophiles) and a remarkable euryhalinism, with strain PN25 showing an optimal growth in the range 35–105‰.

### 3.3 Mycobiota of *Pelagia noctiluca*

Overall, the mycobiota of *P. noctiluca* was mainly composed of Ascomycota (21 taxa; 91.3%) with a small contribution of Basidiomycota (two taxa, 8.7%). Considering the phylum Ascomycota, Dothideomycetes and Eurotiomycetes were the most representative classes with 13 and 5 taxa, respectively. This agrees with previous reports describing these classes as the most representative in marine environments, considering both the number of taxa and abundance (Gonçalves et al., 2022; Wijayawardene et al., 2022).

As for the phylum Basidiomycota, only two taxa (one strain of Tremellomycetes and one of Microbotryomycetes) were identified. Although the result is in line with those previously reported in marine habitats/substrata, it must be also considered that the Basidiomycota could be underestimated due to the use of not selective techniques (Ding et al., 2011, Gonçalves et al., 2022). With respect to the isolation districts, the highest number of species (18) and colonies (89) were associated with the inner tissues, while seven and four species were recovered from the umbrella and oral arms, respectively. Oral arms showed a scarce colonization (27 colonies) and none of the four species found were exclusively associated with this district: *B. caroliniana* and *C. allicinum* were isolated from OA and U, *P. brevicompactum* from OA and IT and *C. delicatulum* was detected in all districts. On the contrary, the 43 and 83% of colonizers recovered in the umbrella and the inner tissues, respectively, were isolated only from one district (Table 2).

The most represented genera, in terms of species, were *Cladosporium* (six species) and *Penicillium* (five species). This result is not surprising since these genera are well adapted to marine environments and appear to be generally ubiquitous in the oceans (Raghukumar, 2017). Some of these species were frequently recorded in several marine habitats and substrata. For example, *P. antarcticum* and *P*. *brevicompactum* are well-known as saprotroph in seawater and as biont/saprotroph of algae, phanerogams, and animals (Paz et al., 2010; López-Legentil et al., 2015; Bovio et al., 2017, 2018, 2019; Marchese et al., 2020, 2021). Other species (i.e., *C*. *aggregatocicatricatum*, *C*. *westerdijkiae*, and *P*. *bialowienzense*) have been recorded on fewer marine substrata, even if they are widespread in terrestrial habitats (Bovio et al., 2017, 2018; Maamar et al., 2020; Ben-Dor Cohen et al., 2021).

Finally, the 22% of taxa (including the new genera and species) were detected for the first time in the marine environment. The high percentage of new strains detected emphasize the gap in our knowledge about real diversity of fungi in the sea. In the meantime, it underlines the great importance to focus on conservation strategies of biodiversity, considering also present and future prospectives linked to dramatic global changes. In this context, the maintaining of marine species in culture collections of microorganisms represents an important tool for their preservation, possible ecosystem restoring, and other applications. Under the ecological point of view, these species might be exclusively associated with *P. noctiluca* and considered as species-specific (“specialized”) organisms (Rambelli et al., 2004). However, the available information regarding the associations of these fungi with jellyfishes and other marine substrata is too scant to allow more than a hypothetical consideration. To the best of our knowledge, *P. noctiluca* was never studied before. Moreover, it is worth noting that most mycological studies on jellyfishes focused only on single strains and their biotechnological potential, without providing any ecological information on the single species or on the whole mycobiota (Wright et al., 2003; Liu et al., 2011, 2012; La Kim et al., 2012a,b; Liu et al., 2013; Yue et al., 2015; Tao et al., 2016; Liu et al., 2018; Regalado and Ramirez, 2019; Tan et al., 2019; Yue et al., 2022; Li et al., 2023). Globally only 14 species belonging to *Cladosporium*, *Purpureocillium*, *Tilletiopsis*, *Aspergillus*, *Epicoccum*, *Paecilomyces*, *Penicillium*, and *Phoma* were collected from the different jellyfish species studied (*A. aurita*, *N. nomurai*, and *Catostylus* sp.) (Supplementary Table S8). However, the only study focusing on a jellyfish mycobiota regarded *N. nomurai*, which showed a relatively low diversity in the fungal assemblage represented by just seven isolates and five species (Yue et al., 2015).

In the present study, 23 different fungal species were found on *P. noctiluca* and none of them was detected in the mycobiota of the other studied jellyfishes. On the contrary, some common genera such as *Cladosporium* and *Penicillium* were observed. Nevertheless, the significance of this genera in the mycobiota jellyfish characterization appears of low relevance considering, as already observed, that species of these genera are generally ubiquitous in the seas. Lastly, it is worth noting that the mycobiota of *P. notiluca* showed a relatively high diversity, if compared to other marine epizoic fungal communities (López-Legentil et al., 2015; Bovio et al., 2019).

### 3.4 Screening for potential bioactive molecules

The potential biotechnological interest of the isolated fungi, within a blue growth strategy perspective, has been tested by both plate screening and molecular survey of the target genes PKS and NRPS. These genes are commonly used to find species of interest for production of bioactive metabolites (Bentley et al., 2002; Molnár et al., 2010) since they are involved in the biosynthesis of a broad range of compounds such as antibiotics, antifungal, antiviral, anticancer, mycotoxins, antifouling, and pigments (Raimundo et al., 2018; Stroe et al., 2024). Moreover, the presence of these genes in fungi associated to marine macro-organisms (i.e., sponges, corals, algae, and mangrove plants) could suggest their potential roles in the host chemical defense process (Zhou et al., 2011; Hafez Ghoran et al., 2023).

A preliminary screening (agar plug diffusion method) was carried out to evaluate the potential production of antimicrobial molecules by the isolated species cultivated on different substrata (Table 3). Different media were utilized to promote different metabolic pathways and metabolites (Pinedo-Rivilla et al., 2022). Globally, the 70% of the strains exhibited inhibitory activity against one or two of the tested microorganisms (*B. pumilus*; *P. fluorescens*; *P*. *griseofulvum*). In general, no activity was observed against *P. fluorescens*, 11 strains showed antibacterial activity against *B. pumilus* and 11 strains antifungal activity. Five strains (PN16, PN31, PN33, PN38, and PN40), were active against both *P*. *griseofulvum* and *B. pumilus* gram-positive bacteria. Strains included in the order Pleosporales (PN9, PN33, PN27, and PN38) showed the strongest antibacterial activity, while the two *Penicillium* strains PN22 (*P. bialowienzense*) and PN25 (*P. brevicompactum*) showed the strongest activity against *P. griseofulvum*. The antifungal activity showed by various fungal species could be due to the production of cell-wall degrading enzymes (i.e., chitinases); however, PN22 and PN25 did not show any chitinolytic activity (data not shown). Considering the different substrata, in general, the most efficient to induce the production of bioactive molecules was PDAs (81% of active strain), only *B. caroliniana* showed activity when growing on all saline media, while only *P. brevicompactum* showed antifungal activity in a salt-free medium (CYA). Even if the antimicrobial activity tested by the plug diffusion method is not exhaustive, this preliminary result confirms that jellyfish-associated fungi can be a good source of active metabolites (Liu et al., 2013; Yue et al., 2015).

**Table 3:** Screening for antimicrobial activity: bioassay of antimicrobial activity of PN strains against *Bacillus pumilus* and *Penicillium griseofulvum*, Culture media, amplification of PKS, and NRPS genes.

Taxon	Antimicrobial activity			Media	PCR amplification	
	*B. pumilus*	*P. griseofulvum*				
	(24 h)	(48 h)	(72 h)		PKS	NRPS
*B. caroliniana* (PN40)	**+**	**+**	**+**	A, B, C	*	n.a.
*B. cinerea* (PN7)	**+++**	**−**	**−**	A	n.a.	*
*C. allicinum* (PN43)	−	−	−	−	n.a.	n.a.
*C. delicatulum* (PN16)	++	+	−	A	n.a.	*
*C. elatum* (PN36)	−	−	−	−	n.a.	n.a.
*C. myrtacearum* (PN3)	−	−	−	−	n.a.	n.a.
*C. halotolerans* (PN17)	++	−	−	A	n.a.	n.a.
*C. westerdijkiae* (PN44)	−	−	−	−	n.a.	n.a.
*Keissleriella* sp. (PN6)	+	−	−	A	n.a.	n.a.
*N. fuckelii* (PN27)	++	−	−	A	n.a.	*
*N. acicola* (PN31)	+	+	+	A	n.a.	*
Phaeosphariaceae sp. (PN9)	+++	−	−	A	n.a.	n.a.
Phaeosphariaceae sp. (PN33)	+++	+	+	A	*	n.a.
*Tamaricicola* sp. (PN38)	++	+	+	A	n.a.	n.a.
*P. antarcticum* (PN35)	−	+	+	A	*	*
*P. bialowienzense* (PN22)	−	+++	+++	C	n.a.	n.a.
*P. brevicompactum* (PN25)	−	+++	+++	D	*	*
*P. fundyense* (PN20)	−	+	+	A	n.a.	*
*P. polonicum* (PN15)	−	+	+	A	n.a.	*
*R. intermedia* (PN1)	++	−	+	A	n.a.	*

−, no activity detected; + (halos of 0–4 mm in diameter); ++ (halos of 5–8 mm in diameter); +++ (halos > 8 mm in diameter); A = PDAs; B = CYAs; C = MEAs; D = CYA; n.a., Not amplified; *amplification. No activity was registered against the Gram-negative *P. fluorescens*.

The results of the molecular survey are generally in accordance with the antimicrobial activity observed with cultural methods. The 70% of active strains presented PKS and/or NRPS genes: NRPS was present in nine strains, while PKS only in four strains and only *P. brevicompactum* and *P. anctarticum* presented both genes. Considering that *Cladosporium* and *Penicillium* were the most representative genera in the *P. noctiluca* fungal assemblage and that the primers used were designed on these taxa, it was quite surprising that the number of positive strains was relatively low. This is particularly true considering that most *Cladosporium* and *Penicillium* strains are known to produce various bioactive compounds related to these genes (El Hajj Assaf et al., 2020; Li et al., 2024). In particular, in this study for the *Cladosporium* genus only *C. delicatulum* was positive to NRPS and none of them to PKS. Finally, it is worth noting that *P. bialowienzense,* showing the strongest antifungal activity, was negative to both genes and, as already observed, to chitinolytic activity; indicating that its biological activity is probably related to others metabolic pathways.

Some new (Phaeosphaeriaceae sp. PN33) or neglected species (i.e., *N. fuckelii* and *R. intermedia*) that have never been studied for the presence of these genes, positive in this study, deserve further investigation.

In addition, the four strain (PN36, PN3, PN44, and PN43) that did not show activity were also negative to the gene amplification. It is possible that these results underestimated the real presence of these target genes, since the primers used for their amplification were designed for other specific genera, i.e., *Penicillium*, *Aspergillus*, and *Cladosporium* (Bingle et al., 1999). Hence, it is possible that they were unable to amplify homologous genes in other genera and species, in particular considering new taxa (Lee et al., 2001).

## 4 Conclusion

This work describes for the first time the culturable mycobiota associated with the jellyfish *Pelagia noctiluca* and highlights the relatively high biodiversity of marine fungi associated with this jellyfish. In addition, the study evidenced the presence of several marine strains, which were detected for the first time in the sea environment. Moreover, the identification of new taxa enphasize the gap in our knowledge about the real entity of the fungal impact in the sea. In the meantime, the work underlines the great importance to focus the research on conservation strategies of the fungal biodiversity, considering also the prospectives linked to rapidly increasing global changes. In this context, the conservation of marine strains in culture collections of microorganisms, according to the most recent good practices, represents an important strategy for their preservation, possible ecosystem restoring, and a number of potential applications also in the One Health framework. Here, the new marine strains appeared of particular interest for blue-biotechnological perspectives, since the screening results suggested that some of them could be used for the production of new drugs. However, further investigations need to be performed to evaluate the actual biotechnological value of the studied strains. This work evidenced the potential of the epizoic fungi related to *P. noctiluca* and lead the way to further investigations, not only to formally describe the new taxa, but also to get a deeper picture of the whole microbiota of this jellyfish, including molecular studies to depict the non-culturable moiety of the community. Studies in this sense are already in progress.

## Data Availability

The datasets presented in this study can be found in online repositories. The names of the repository/repositories and accession number(s) can be found in the article/Supplementary material.
