# Supplementary material for: From marine neglected substrata new fungal taxa of potential biotechnological interest: the case of Pelagia noctiluca
Source: Front Microbiol. 2024 Oct 11;15:1473269. doi: 10.3389/fmicb.2024.1473269 (PMC11502404; doi:10.3389/fmicb.2024.1473269)
Supplement: Supplementary file 1 [file Data_Sheet_1.ZIP › Supplementary Materials1/Pasqualetti et al._Frontiers_SUPPLEMENTARY MATERIAL.docx]

Supplementary Material

Table S1. Taxa included in the dataset used in the phylogenetic analysis (nrITS region) and GenBank accession number. Newly generated sequences are indicated in bold.

| **Species** | **Strain** | **GenBank AN** |
| --- | --- | --- |
| *Aaosphaeria arxii* | CBS 175.79 | MH861193 |
|  | MUT <ITA>:5333 | KU158145 |
| *Alternaria* sp. | 174wat | KF811432 |
|  | DoF22 | JQ388267 |
| *Arthopyrenia salicis* | UTHSC: DI16-356 | LT766900 |
|  | UTHSC:DI16-220 | LT796841 |
| *Botrytis caroliniana* | CH_WS_11 | MT548671 |
|  | **PN40** | **ON807238** |
| *Botrytis cinerea* | BCH04 | KU992696 |
|  | BC201998 | MN420829 |
|  | Bc28L-18Ps | MN186739 |
|  | **PN02** | **OP793783** |
|  | **PN07** | **ON807239** |
| *Botrytis cinerea* (*Botryotinia fuckeliana*) | 192B0 | HM849615 |
| *Botrytis elliptica* | LBM137423 | KR055047 |
| *Botrytis fabae* | T25-2 | KC213772 |
| *Botrytis tulipae* | CBS 286.71 | MH860126 |
| *Chaetomium elatum* | isolate 76 | MN534823 |
|  | **PN36** | **MZ222288** |
| *Chaetomium globosum* | E-824 | KU059939 |
| *Chaetomium rectangulare* | CBS 126778 | MH864225 |
| *Cladosporium aggregatocicatricatum* | MUT <ITA>: 2524 | MG813175 |
|  | **PN29** | **MZ221935** |
| *Cladosporium allicinum* | isolate 9 | MT573471 |
|  | **PN43** | **MZ221774** |
|  | **PN10** | **OP793782** |
| *Cladosporium austroafricanum* | 2018A0762M00 | MT378414 |
| *Cladosporium cladosporioides* | 9Y_G10 | MT131338 |
| *Cladosporium delicatulum* | CL_FF_10_con | MT548673 |
|  | DTO:305-I9 | MF472954 |
|  | **PN16** | **MZ221944** |
|  | **PN26** | **OP793881** |
|  | **PN42** | **OP793885** |
|  | **PN08** | **OP793794** |
|  | **PN12** | **OP793841** |
|  | **PN19** | **OP793882** |
|  | **PN11** | **OP793798** |
| *Cladosporium halotolerans* | DTO:147-B8 | MF473012 |
|  | **PN17** | **MZ221974** |
| *Cladosporium herbarum* | YMZZ2 | MN486496 |
| *Cladosporium limoniforme* | C19 | MK818537 |
| *Cladosporium myrtacearum* | **PN03** | **MZ221760** |
| *Cladosporium oxysporum* | SS_26 | MT497419 |
| *Cladosporium perangustum* | CL2 | MT466522 |
| *Cladosporium westerdijkiae* | **PN44** | **MZ222249** |
| *Keissleriella cladophila* | CBS 104.55 | MH857391 |
| *Keissleriella rosarum* | MFLUCC15-0089 | MG828905 |
| *Keissleriella* sp. | **PN6** | **ON807306** |
| *Lophiostoma macrostomum* | CBS:122680 | EU552140 |
| *Leptosphaeria* sp. | Z.-Y-40 | MK367481 |
| *Neovaginatispora fuckelii* | KH161 | LC001731 |
|  | SICAUCC 20-D008 | MT427731 |
|  | **PN27** | **ON807307** |
| *Neopyrenochaeta acicola* | CBS 812.95 | LT623218 |
|  | **PN31** | **ON806621** |
| *Papiliotrema anemochoreia (Cryptococcus anomochoreius*) | WM 13.144 | KP068781 |
| *Papiliotrema fonsecae* (*Cryptococcus fonsecae*) | DSM 26992 | LK023835 |
| *Papiliotrema fonsecae* | AMF008 | MN309710 |
| *Papiliotrema laurentii* | R97299 | MK268144 |
| *Papiliotrema* sp. | **PN21** | **MZ254988** |
| *Phaeosphaeria graminis* | CBS:123087 | MH863271 |
| *Penicillium antarcticum* | CBS 100492 | MH862703 |
|  | **PN35** | **MZ254898** |
|  | **PN14** | **OP793893** |
| *Penicillium attenuatum* | GBC_Fungus 94 | MN077471 |
| *Penicillium bialowiezense* | CBS 227.28 | NR_165994 |
|  | **PN22** | **MZ254911** |
| *Penicillium biourgeianum* | RP114_36 | KX067821 |
| *Penicillium brevicompactum* | CBS 287.53 | MH857204 |
|  | isolate 654 | DQ682592 |
|  | **PN25** | **MZ254900** |
|  | **PN37** | **OP793895** |
|  | **PN13** | **OP793896** |
| *Penicillium fundyense* | **PN20** | **MZ254909** |
|  | **PN24** | **OP793891** |
| *Penicillium herquei (P. coralligerum)* | CBS 114.69 | KP016836 |
| *Penicillium cordubense* | G8PI_P8 | KP712867 |
| *Penicillium kongii* | AS3.15329 | NR_138336 |
| *Penicillium lapidosum* | ssw | KJ676451 |
| *Penicillium polonicum* | E20379 | MK267441 |
|  | **PN15** | **MZ254904** |
| *Phaeosphaeria* cf. *caricicola* | 2015.371 | MH399340 |
| *Phaeosphaeria* sp. | isolate P2410 | KT269643 |
|  | A1.T12 | MH935028 |
| *Phaeosphaeriaceae* sp. | **PN9** | **ON807308** |
|  | **PN33** | **ON807308** |
| Pleosporales sp. | E-000535891 | JN545826 |
|  | MU-2009-2 | FN548155 |
| *Roussoella intermedia* | **PN1** | **ON805845** |
| *Roussoella intermedia* | CBS 170.96 | KF443407 |
| *Roussoella neopustulans* | FJI L9 BK P1 | MT704872 |
| *Sporobolomyces roseus* | CBS: 5541 | KY105476 |
|  | AUMC 11209 | KY495777 |
|  | **PN4** | **MZ221766** |
| *Tamaricicola muriformis* | IT_9172 | KU900317 |
| *Tamaricicola* sp. | IG114 | MG977427 |
|  | **PN38** | **ON807355** |
|  | **PN39** | **OP794025** |
|  | **PN23** | **OP793911** |
|  | **PN28** | **OP793912** |
|  | **PN32** | **OP793995** |

Table S2. Taxa included in the dataset used in the phylogenetic analysis based on *actin* gene. The GenBank Accession Number was also reported.

| **Species** | **Strain** | **GenBank AN** |
| --- | --- | --- |
| *Cladosporium aggregatocicatricatum* | i36 | MG680499 |
|  | i37 | MG680500 |
|  | MUT <ITA>:2524 | MG832112 |
|  | **PN29** | **MZ305182** |
| *Cladosporium allicinum* | IG124 | MG066651 |
|  | i21 | MG680501 |
|  | CPC:11840 | EF679497 |
|  | MUT <ITA>:3724 | MK050511 |
|  | **PN43** | **MZ305181** |
|  | UTHSC DI-13-176 | LN834538 |
| *Cladosporium asperulatum* | BP8I3 | KU605799 |
|  | BP8I2 | KU605798 |
| *Cladosporium delicatulum* | DETSC02 | KT887878 |
|  | DTO:305-H7 | MF473803 |
|  | CBS 126342 | HM148568 |
|  | CBS 126343 | HM148569 |
|  | **PN16** | **MZ305184** |
| *Cladosporium halotolerans* | MUT <ITA>:1299 | KR856500 |
|  | UTHSC DI-13-182 | LN834551 |
|  | FMR 13493 | LN834549 |
|  | DTO:305-F3 | MF473873 |
|  | MUT 3271 | MH047333 |
|  | **PN17** | **MZ305183** |
| *Cladosporium myrtacearum* | CBS126350 | HM148606 |
|  | CLAD150 | MK314669 |
|  | CBS126349 | HM148605 |
|  | **PN3** | **MZ305185** |
| *Cladosporium pseudocladosporioides* | is3 | MG680535 |
|  | is4 | MG680536 |
| *Cladosporium westerdijkiae* | CBS113746 | HM148548 |
|  | CPC14284 | HM148552 |
|  | DTO:152-A9 | MF474163 |
|  | CPC13362 | HM148550 |
|  | CPC 13978 | HM148551 |
|  | **PN44** | **MZ291568** |
| *Cladosporium* sp. | MUT 1730 | KU935620 |
| **Outgroup** |  |  |
| *Cercospora beticola* | WY4-4 | MF680965 |

Table S3. Taxa included in the dataset used in the phylogenetic analysis based on *β-tubulin* gene. The GenBank Accession Number was also reported.

| **Species** | **Strain** | **GenBank AN** |
| --- | --- | --- |
| *Penicillium antarcticum* | 24E | MK519554 |
|  | JM0840 | KU600412 |
|  | MUT <ITA>:5201 | KT779542 |
|  | MUT <ITA>:1797 | KU935633 |
|  | MUT <ITA>:2250 | MG832184 |
|  | MUT <ITA>:1805 | KU935643 |
|  | **PN35** | **ON866496** |
| *Penicillium bialowiezense* | FC092 | KT588891 |
|  | CMV009C2 | MK451151 |
|  | AS3.15321 | KC427163 |
|  | AS3.15314 | KC427156 |
|  | **PN22** | **ON866500** |
| *Penicillium biourgeianum* | NRRL 32207 | DQ645792 |
|  | NRRL 32305 | DQ645791 |
| *Penicillium brevicompactum* | MUT <ITA>:5036 | MN173610 |
|  | SCCM 10-A6 | EU587358 |
|  | MUT <ITA>:4965 | KT313384 |
|  | MUT <ITA>:5169 | KT779535 |
|  | MUT3501 | MH047311 |
|  | **PN25** | **ON866497** |
| *Penicillium citreonigrum* | BEOFB11190m | MH780073 |
|  | MUT <ITA>:2062 | MG832162 |
|  | DI16-86 | LT559026 |
| *Penicillium fundyense* | KAS2174 | KT887814 |
|  | **PN20** | **ON866499** |
| *Penicillium polonicum* | YC-IK10 | MK862427 |
|  | KAS5979 | KY469168 |
|  | G5314 | KF021549 |
|  | DI16-102 | LT559042 |
|  | **PN15** | **ON866498** |
| *Penicillium* sp. | GZU-BCECD56 | JQ965088 |
| **Outgroup** |  |  |
| *Aspergillus terreus* | isolate 1549 | MF185073 |

Table S4. Taxa included in the concatenate dataset used in the multi-locus phylogenetic analysis based on G3PDH, HSP60, *rpb-*2 genes. The GenBank Accession Number was also reported.

| Species | Strain | GenBank AN | | |
| --- | --- | --- | --- | --- |
|  |  | **G3PDH** | **HSP60** | ***rpb-2*** |
| *Botrytis aclada* | MUCL 3106 | AJ704991 | AJ716049 | AJ745663 |
|  | MUCL 8415 | AJ704992 | AJ716050 | AJ745664 |
|  | PRI 006 | AJ704993 | AJ716051 | AJ745665 |
| *Botrytis allii* | MUCL 403 | AJ704997 | AJ716058 | AJ745667 |
| *Botrytis byssoidea* | MUCL 94 | AJ704998 | AJ716059 | AJ745670 |
| *Botrytis californica* | X655 | KJ937069 | KJ937059 | KJ937049 |
| *Botrytis calthae* | CBS175.63 | AJ704999 | AJ716060 | AJ745671 |
|  | MUCL2830 | AJ705001 | AJ716062 | AJ745673 |
|  | MUCL1089 | AJ705000 | AJ716061 | AJ745672 |
| *Botrytis caroliniana* | CB 15 | - | - | JF811590 |
|  | **PN40** | **OP019701** | **OP019699** | **ON887326** |
| *Botrytis cinerea* | BC-1 | EU563103 | EU563096 | EU563113 |
|  | BC-15 | EU563108 | EU563099 | EU563114 |
|  | BC-27 | EU563107 | EU563101 | EU563118 |
|  | B05.10 | JQ036050 | JQ036108 | - |
|  | **PN7** | **OP019700** | **OP019698** | **ON887325** |
| *Botrytis convoluta* | 9801 | AJ705007 | AJ716068 | AJ745679 |
|  | MUCL11595 | AJ705008 | AJ716069 | AJ745680 |
| *Botrytis croci* | MUCL 436 | AJ705009 | AJ716070 | AJ745681 |
| *Botrytis deweyae* | CBS134649 | HG799521 | HG799519 | HG799518 |
| *Botrytis elliptica* | BE9714 | AJ705012 | AJ716073 | AJ745684 |
|  | BE9610 | AJ705011 | AJ716072 | AJ745683 |
|  | BE0022 | AJ705010 | AJ716071 | AJ745682 |
| *Botrytis eucalypti* | CERC7170 | KX301020 | KX301024 | KX301028 |
| *Botrytis euroamericana* | HA06 | KX266728 | KX266734 | KX266740 |
| *Botrytis fabae* | BC-17 | EU563104 | EU563095 | EU563112 |
|  | BC-22 | EU563105 | EU563102 | EU563111 |
|  | BC-25 | EU563110 | EU563098 | EU563116 |
|  | MUCL98 | AJ705014 | AJ716075 | AJ745686 |
| *Botrytis fabiopsis* | BC-13 | EU563109 | EU563100 | EU563115 |
|  | BC-30 | EU563106 | EU563097 | EU563117 |
|  | BC-2 | EU519211 | EU514482 | EU514473 |
| *Botrytis ficariarum* | CBS176.63 | AJ705015 | AJ716076 | AJ745687 |
|  | MUCL376 | AJ705016 | AJ716077 | AJ745688 |
| *Botrytis fragariae* | U14P1 | KX429699 | KX429692 | KX429706 |
| *Botrytis galanthina* | MUCL 435 | AJ705018 | AJ716079 | AJ745689 |
|  | MUCL 3204 | AJ705017 | AJ716078 | AJ745690 |
| *Botrytis gladiolorum* | 9701 | AJ705019 | AJ716080 | AJ745691 |
|  | MUCL 3865 | AJ705020 | AJ716081 | AJ745692 |
| *Botrytis globosa* | MUCL 444 | AJ705022 | AJ716083 | AJ745693 |
|  | MUCL 21514 | AJ705021 | AJ716082 | AJ745694 |
| *Botrytis hyacinthi* | MUCL 442 | AJ705024 | AJ716085 | AJ745696 |
|  | 0001 | AJ705023 | AJ716084 | AJ745695 |
| *Botrytis medusae* | B-555 | MH732861 | MH732866 | MH732870 |
| *Botrytis narcissicola* | MUCL 18857 | AJ705025 | AJ716086 | AJ745698 |
|  | MUCL 2120 | AJ705026 | AJ716087 | AJ745697 |
| *Botrytis paeoniae* | MUCL16084 | AJ705028 | AJ716089 | AJ745700 |
|  | 0003 | AJ705027 | AJ716088 | AJ745699 |
| *Botrytis pelargonii* | CBS497.50 | AJ704990 | AJ716046 | - |
|  | MUCL1152 | AJ705029 | AJ716090 | AJ745701 |
| *Botrytis polyblastis* | CBS287.38 | AJ705030 | AJ716091 | AJ745702 |
|  | MUCL 21492 | AJ705031 | AJ716092 | AJ745703 |
| *Botrytis polyphylla* | YN1 | MG387957 | MG431978 | MG431975 |
| *Botrytis porri* | MUCL3234 | AJ705032 | AJ716093 | AJ745704 |
|  | MUCL3349 | AJ705033 | AJ716094 | AJ745705 |
| *Botrytis prunorum* | Bpru 1.5 | KP339984 | KP339998 | KP339991 |
| *Botrytis pseudocinerea* | 10091 | JN692414 | JN692400 | JN692428 |
| *Botrytis pyriformis* | SEdsarBC1 | KJ543484 | KJ543488 | KJ543492 |
| *Botrytis ranunculi* | CBS178.63 | AJ705034 | AJ716095 | AJ745706 |
| *Botrytis sinoallii* | Onion BC-23 | EU519217 | EU514488 | EU514479 |
| *Botrytis sinoviticola* | GBC-5 | JN692413 | JN692399 | JN692427 |
| *Botrytis sphaerosperma* | MUCL21481 | AJ705035 | AJ716096 | AJ745708 |
|  | MUCL21482 | AJ705036 | AJ716097 | AJ745709 |
| *Botrytis squamosa* | PRI026 | AJ705039 | AJ716100 | AJ745707 |
|  | MUCL1107 | AJ705037 | AJ716098 | AJ745710 |
|  | MUCL9112 | AJ705038 | AJ716099 | AJ745711 |
| *Botrytis tulipae* | BT9901 | AJ705042 | AJ716103 | AJ745714 |
|  | BT9830 | AJ705041 | AJ716102 | AJ745713 |
|  | BT9001 | AJ705040 | AJ716101 | AJ745712 |
| Outgroup |  |  |  |  |
| *Sclerotinia sclerotiorum* | 1980 | JQ036048 | JQ036098 | - |

Table S5. Taxa included in the concatenate dataset used in the multi-locus phylogenetic analysis of the order Pleosporales based on nrLSU, nrSSU, nrITS markers. The GenBank Accession Number was also reported.

| **Species** | **Strains** | **GenBank AN** | | |
| --- | --- | --- | --- | --- |
|  |  | **nrLSU** | **nrSSU** | **nrITS** |
| *Aaosphaeria arxii* | CBS 175.79 | - | MH872962 | MH861193 |
| *Aigialus grandis* | JK 5244A | GU301793 | GU296131 | - |
| *Aigialus mangrovis* | BCC 33563 | GU479776 | GU479741 | - |
| *Aigialus parvus* | BCC 18403 | GU479778 | GU479744 | - |
| *Alternaria maritima* | CBS 126.60 | MH869463 | GU456294 | - |
| *Arthopyrenia salicis* | UTHSC:DI16-220 | LN907363 | - | LT796841 |
| *Ascochyta pisi* | CBS 126.54 | MH868800 | EU754038 | MH857263 |
| *Bambusicola bambusae* | MFLUCC 11-0614 | JX442035 | JX442039 | JX442031 |
| *Bambusicola irregulispora* | MFLUCC 11-0437 | JX442036 | JX442040 | JX442032 |
| *Bambusicola massarinia* | MFLUCC 11-0389 | JX442037 | JX442041 | JX442033 |
| *Bambusicola splendida* | MFLUCC 11-0439 | JX442038 | JX442042 | JX442034 |
| *Biatriospora marina* | CY_1228 | GQ925848 | GQ925835 | - |
| *Biatriospora* sp. | MUT 4407 | KF636783 | - | KC339242 |
| *Bimuria novae-zelandiae* | CBS 107.79 | MH872950 | NG_061017 | MH861181 |
| *Byssosphaeria jamaicana* | SMH 1403 | GU385152 | - | - |
| *Byssosphaeria schiedermayeriana* | GKM 152N | GU385168 | - | - |
| *Byssosphaeria villosa* | GKM 204N | GU385151 | - | - |
| *Byssothecium circinans* | CBS 675.92 | AY016357 | GU205235 | OM337536 |
| *Cochliobolus heterostrophus* | AFTOL-ID 54 | AY544645 | AY544727 | DQ491489 |
| *Cochliobolus sativus* | AFTOL-ID 271 | DQ678045 | DQ677995 | - |
| *Cucurbitariaceae* sp. | MUT 4403 | KF636780 | - | KC339238 |
| *Didymella cucurbitacearum* | IMI 373225 | AY293792 | AY293779 | AY293804 |
| *Didymella vitalbina* | CBS 123706 | FJ515635 | - | FJ515594 |
| *Didymellaceae* sp. | MUT 4313 | KF636771 | - | KC339219 |
| *Dothidotthia aspera* | CPC 12932 | EU673275 | EU673227 | - |
| *Dothidotthia symphoricarpi* | CPC 12929 | EU673273 | EU673224 | - |
| *Herpotrichia juniperi* | CBS 200.31 | MH866637 | - | - |
| *Hysterium angustatum* | CBS:236.34 | FJ161180 | GU397359 |  |
| *Hysterobrevium baoshanense* | MFLUCC 16-2162 | KX772765 | KX772767 | MZ467049 |
| *Hysterobrevium smilacis* | CBS:114601 | FJ161174 | FJ161135 | |
| *Kalmusia scabrispora* | KT 2202 | AB524594 | AB524453 | - |
| *Katumotoa bambusicola* | KT 1517a | AB524595 | AB524454 | LC014560 |
| *Keissleriella cladophila* | CBS 104.55 | JX681090 | GU296155 | MH857391 |
| *Keissleriella dactylis* | MFLUCC 13-0751 | KP197668 | KP197666 | KP197667 |
| *Keissleriella* sp. | **PN6** | **ON838727** | **ON839999** | **ON807306** |
| *Lentitheciaceae* sp. | MUT 4420 | KF636787 | - | KF636770 |
| *Lentithecium arundinaceum* | CBS 619.86 | GU301824 | GU296157 | - |
| *Leptosphaeria biglobosa* | CBS 303.51 | MH868390 | - | AJ550892 |
| *Leptosphaeria maculans* | AFTOL-ID 277 | DQ470946 | DQ470993 | KT225526 |
| *Leptosphaerulina australis* | CBS 939.69 | MH871269 | EU754068 | MH859488 |
| *Lophiostoma arundinis* | AFTOL-ID 1606 | DQ782384 | DQ782383 | - |
| *Lophiostoma crenatum* | AFTOL-ID 1581 | DQ678069 | DQ678017 | - |
| *Lophiostoma sagittiforme* | HHUF 29754 | NG_042319 | - | NR_119393 |
| *Lophiostoma scabridisporum* | BCC 22835 | GQ925844 | GQ925831 | - |
| *Loratospora aestuari* | [CBS 117592](https://www.ncbi.nlm.nih.gov/nuccore/MH863024.1) | MH874575 | - | MH863024 |
| *Massarina cisti* | CBS 266.62 | FJ795447 | AB797249 | - |
| *Massarina eburnea* | CBS 473.64 | FJ795449 | GU296170 | OM337528 |
| *Melanomma pulvis-pyrius* | CBS 109.77 | FJ201986 | FJ201987 | OM238167 |
| *Misturatosphaeria claviformis* | GKM 1210 | GU385212 | - | - |
| *Misturatosphaeria tennesseensis* | ANM 911 | GU385207 | - | - |
| *Misturatosphaeria uniseptata* | SMH 4330 | GU385167 | - | - |
| *Montagnula opulenta* | AFTOL-ID | DQ678086 | - | - |
| *Neopyrenochaeta acicola* | MUT 4382 | KJ395497 | - | KJ395501 |
|  | CBS 812.95T | GQ387602 | NG_065567 | LT623218 |
|  | **PN31** | - | - | **ON806621** |
| *Neopyrenochaeta fragariae* | CBS 101634 | GQ387603 | GQ387542 | LT623217 |
| *Neopyrenochaeta telephoni* | CBS 139022T | MH877672 | KR260987 | - |
| *Neosetophoma samarorum* | CBS138.96 | MH874195 | GQ387517 | MH862569 |
| *Neovaginatispora fuckelii* | CBS 101952 | DQ399531 | FJ795496 | - |
|  | KH 161 | AB619008 | AB618689 | LC001731 |
|  | KT 634 | AB619009 | AB618690 | LC001732 |
|  | **PN27** | **ON838938** | **ON840004** | **ON807307** |
| *Nigrograna mackinnonii* | CBS 674.75 | GQ387613 | GQ387552 | KF015654 |
|  | CBS 110022 | GQ387614 | GQ387553 | KF015653 |
| *Paraconiothyrium fuscomaculans* | CBS 116.16 | EU754197 | EU754098 | MH854649 |
| *Paraconiothyrium minitans* | CBS 122786 | EU754174 | EU754075 | - |
| *Paraconiothyrium tiliae* | CBS 265.94 | EU754139 | EU754040 | - |
| *Parastagonospora avenae* | CBS 290.69 | KF251679 | - | MH859307 |
|  | CBS 289.69 | KF251679 | - | KF251174 |
| *Parastagonospora nodorum* | CBS 110109 | MH874441 | EU754076 | KF251177 |
| *Parastagonospora poae* | CBS 135089 | KF251682 | - | - |
|  | CBS 135091 | KF251683 | - | KF251178 |
| *Parathyridaria ramulicola* | MUT 4397 | KF636775 | KC339235 | - |
| *Parathyridariella dematiacea* | MUT 4419 | KF636786 | KC339245 | - |
| *Phaeodothis winteri* | CBS 182.58 | GU301857 | GU296183 | - |
| *Phaeosphaeria nigrans* | CBS 576.86 | GU456331 | - | MH861993 |
| *Phaeosphaeria nodorum* | Sn37-1 | EF590318 | EU189213 | - |
| *Phaeosphaeria olivacea* | JK 5540Q | GU479807 | - | - |
| *Phaeosphaeria spartinicola* | JK 5177A | GU479808 | - | - |
| *Phaeosphaeriaceae* sp. | MUT 4404 | KF636781 | - | KC339239 |
|  | **PN9** | **ON838726** | **ON840001** | **ON807308** |
|  | **PN33** | **ON838942** | **ON840006** | **ON807308** |
| *Phoma cladoniicola* | CBS 128025 | JQ238625 | JQ238623 | KP170641 |
| *Phoma foliaceiphila* | strain FL3 | JQ318010 | - | JQ318008 |
| *Phoma herbarum* | CBS 615.75 | KF251715 | EU754087 | KF251212 |
| *Pleospora herbarum* | CBS 191.86 | JX681120 | GU238232 | MH861935 |
| *Pleospora sedicola* | CBS 109843 | AY849958 | KU850571 | MH862839 |
| *Pleospora typhicola* | MUT 4379 | KF636774 | - | KF636768 |
|  | CBS 132.69 | JF740325 | JF740105 | MH859275 |
| *Pleurophoma pleurospora* | CBS 116668 | JF740326 | - | - |
| *Preussia funiculata* | CBS 659.74 | GU301864 | GU296187 | - |
| *Preussia lignicola* | CBS 264.69 | GU301872 | GU296197 | - |
| *Preussia minima* | AFTOL-ID 1256 | DQ678056 | DQ678003 | - |
| *Preussia terricola* | AFTOL-ID 282 | AY544686 | - | KT225529 |
| *Prosthemium canba* | KT2229C | AB553765 | - | AB554095 |
| *Prosthemium orientale* | KT2088-1 | AB553749 | - | AB554080 |
| *Lophiostoma scabridisporum* | BCC 22835 | GQ925844 | GQ925831 - |  |
| *Pyrenochaeta cava* | CBS 115979 | EU754198 | EU754099 | MH862993 |
| *Pyrenochaeta quercina* | CBS 297.74 | GQ387620 | EU754078 | LT623221 |
| *Pyrenochaeta* sp. | MUT 4378 | KF636773 | KF636767 | KC339228 |
| *Pyrenochaeta unguis-hominis* | CBS 111112 | GQ387623 | GQ387562 | - |
| *Pyrenochaetopsis decipiens* | CBS 165.89 | GQ387625 | GQ387564 | - |
| *Pyrenochaetopsis indica* | CBS 124454 | GQ387626 | GQ387565 | NR_160058 |
| *Pyrenochaetopsis leptospora* | CBS 101635 | GQ387627 | NG_063097 | MH862744 |
| *Pyrenochaetopsis microspora* | CBS 101333 | GQ387630 | GQ387569 | - |
| *Pyrenochaetopsis* sp. | MUT 4273 | KJ395496 | - | KJ395500. |
| *Pyrenophora tritici-repentis* | AFTOL-ID 173 | AY544672 | AY544716 | - |
| *Roussoella hysterioides* | KT 1651 | AB524621 | AB524480 | - |
| *Roussoella intermedia* | CBS 170.96 | KF443382 | KF443390 | KF443407 |
|  | **PN1** | - | **ON839998** | **ON805845** |
| *Roussoella pustulans* | KT 1709 | AB524623 | AB524482 | - |
| *Roussoellopsis tosaensis* | KT 1659 | AB524625 | AB524484 | - |
| *Stagonosporopsis dorenboschii* | CBS 320.90 | GU238184 | - | GU237830 |
| *Stagonosporopsis heliopsidis* | PD 95/6189 | GU238187 | - | GU237924 |
| *Stagonosporopsis loticola* | CBS 562.81 | GU238192 | - | MH861379 |
| *Tamaricicola muriformis* | IT_9173 | KU561879 | KU870909 | KU752187 |
|  | IT_9174 | KU729857 | KU870910 | KU752188 |
|  | IT_9175 | KU729856 | KU870911 | KU752189 |
| *Tamaricicola* sp. | IG108 | MG976984. | MG976980 | MG977425 |
|  | **PN38** | **ON807355** | - | - |
| *Tingoldiago graminicola* | KH 68 | AB521743 | AB521726 | - |
| *Westerdykella angulata* | CBS 610.74 | NG_057754 | NG_062146 | NR_155956 |
| *Westerdykella capitulum* | CBS 337.65 | GU238054 | GU238212 | MH858595 |
| *Westerdykella cylindrica* | CBS 454.72 | NG_027595 | - | - |

Table S6. Genetic distance (percentages of similarity and query coverage) between the sequences of Phaeosphaeriaceae sp. PN33 and the closest genera/species for all markers analyzed.

| **Species (strains)** | **Molecular markers** | | | | |
| --- | --- | --- | --- | --- | --- |
|  | **nrITS** | **nrLSU** | **nrSSU** | ***tef1-α*** | ***rpb*-2** |
| *Neosetophoma samarorum* (CBS 138.96) | 91.83 (83) | 99.15 (99) | 99.81 (100) | - | - |
| *Leptospora thailandica* (MFLUCC 16-0385) | 88.96 (86) | 98.94 (65) | 99.90 (96) | 94.09 (99) | - |
| *Leptospora rubella* (CPC 11006) | 88.77 (83) | 99.16 (64) | 99.9 (100) | - | - |
| *Leptospora galii* (KUMCC 15-0521) | 89.81 (86) | 98.78 (63) | 99.65 (86) | 95.95 (95) | - |
| *Ophiosphaerella agrostidis* (MFLUCC 12-0007) | 91.78 (80) | 98.29 (63) | 99.9 (100) | 95.22 (91) | 84.53 (76) |
| *Ophiosphaerella aquatica* (MFLUCC 14-0033) | 92.16 (86) | 98.09 (76) | 99.9 (100) | 95.08 (95) | - |
| *Equiseticola fusispora* (MFLUCC 14-0522) | 89.29 (80) | 98.90 (63) | 99.80 (100) | 92.78 (95) | - |
| *Phaeosphaeria musae* (MFLUCC 11-0015) | 89.68 (86) | 99.75 (61) | 99.08 (97) | 95.56 (91) | - |
| *Brunneomurispora lonicerae* (KUMCC 18-0158) | 92.00 (78) | 99.12 (61) | 99.9 (98) | 95.97 (90) | - |
| Phaeosphaeriaceae sp. PN9 | 88.3 (83) | 98.80 (96) | 98.98 (98) | 92.64 (98) | - |

Table S7. Genetic distance (percentages of similarity and query coverage) between the sequences of Phaeosphaeriaceae sp. PN9 and the closest genera/species for all markers analyzed.

| **Species (strains)** | **Molecular markers** | | | | |
| --- | --- | --- | --- | --- | --- |
|  | **nrITS** | **nrLSU** | **nrSSU** | ***tef1-α*** | ***rpb*-2** |
| *Leptospora thailandica* (MFLUCC 16-0385) | 86.42 (83) | 98.06 (65) | 98.87 (97) | 92.24 (96) | - |
| *Leptospora rubella* (CPC 11006) | 86.92 (80) | 98.40 (65) | 98.89 (99) | - | - |
| *Leptospora galii* (KUMCC 15-0521) | 86.54 (83) | 98.62 (63) | 98.62 (87) | 94.53 (95) | - |
| *Populocrescentia ammophilae* (MFLUCC 17-0665) | 84.50 (85) | 97.25 (64) | 98.88 (100) | 93.04 (95) | - |
| *Populocrescentia forlicesenensis* (MFLUCC 14-0651) | 90.62 (68) | 98.64 (64) | 98.89 (98) | 93.30 (79) | - |
| *Ophiosphaerella agrostidis* (MFLUCC 12-0007) | 88.37 (69) | 97.74 (63) | 98.90 (100) | 91.87 (90) | 82.06 (65) |
| *Ophiosphaerella aquatica* (MFLUCC 14-0033) | 86.13 (85) | 97.85 (78) | 98.89 (98) | 91.36 (95) | - |
| Equiseticola fusispora (MFLUCC 14-0522) | 87.10 (67) | 98.37 (63) | 98.79 (99) | 89.28 (95) |  |
| *Neosetophoma samarorum* (CBS 138.96) | 85.59 (80) | 98.80 (100) | 98.69 (99) | - |  |
| *Phaeosphaeria musae* (MFLUCC 11-0015) | 86.32 (84) | 98.46 (62) | 98.07 (98) | 94.04 (90) | - |
| *Embarria clematidis* (MFLUCC 14-0976) | 87.55 (83) | 97 (64) | 98.99 (99) | 93.43 (95) |  |
| *Embarria clematidis* (MFLUCC 14-0652) | 89.27 (69) | 97.02 (64) | 98.48 (98) |  |  |
| Phaeosphaeriaceae sp. PN33 | 88.32 (83) | 98.80 (100) | 98.98 (98) | 92.64 (97) |  |


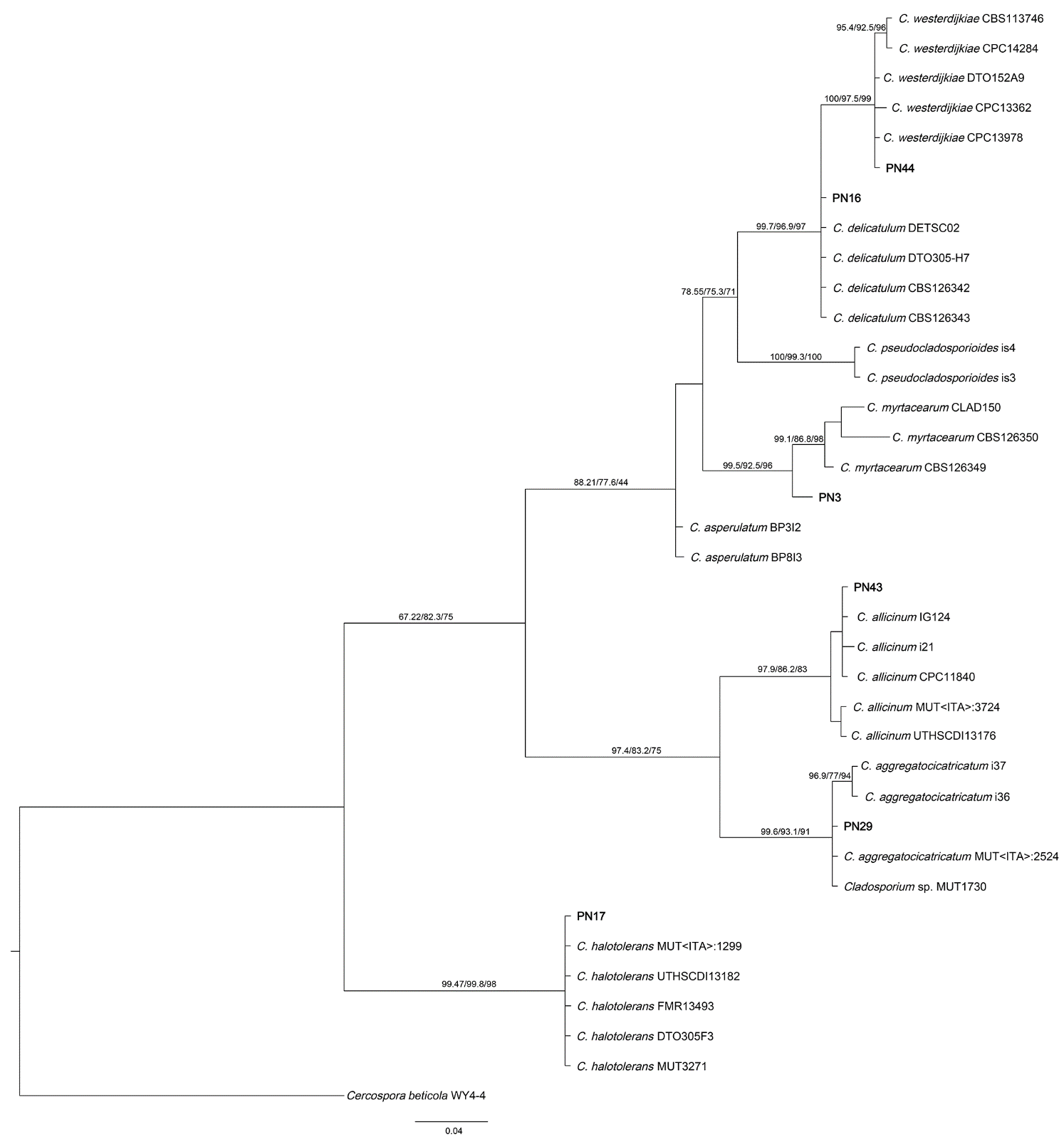


Figure S1. *Cladosporium*: Phylogenetic inference based on *actin.* Dataset was inferred using the Bayesian method*, Cercospora beticola* was utilized as outgroup. Branch numbers indicate BYPP (Bayesian posterior probabilities), and sH-aLRT, and BP values from RA-ML analysis. The Bayesian Inference was performed under model as evaluated by jModelTest 2 using Bayesian Information Criterion (HKY+G, G=0.390).


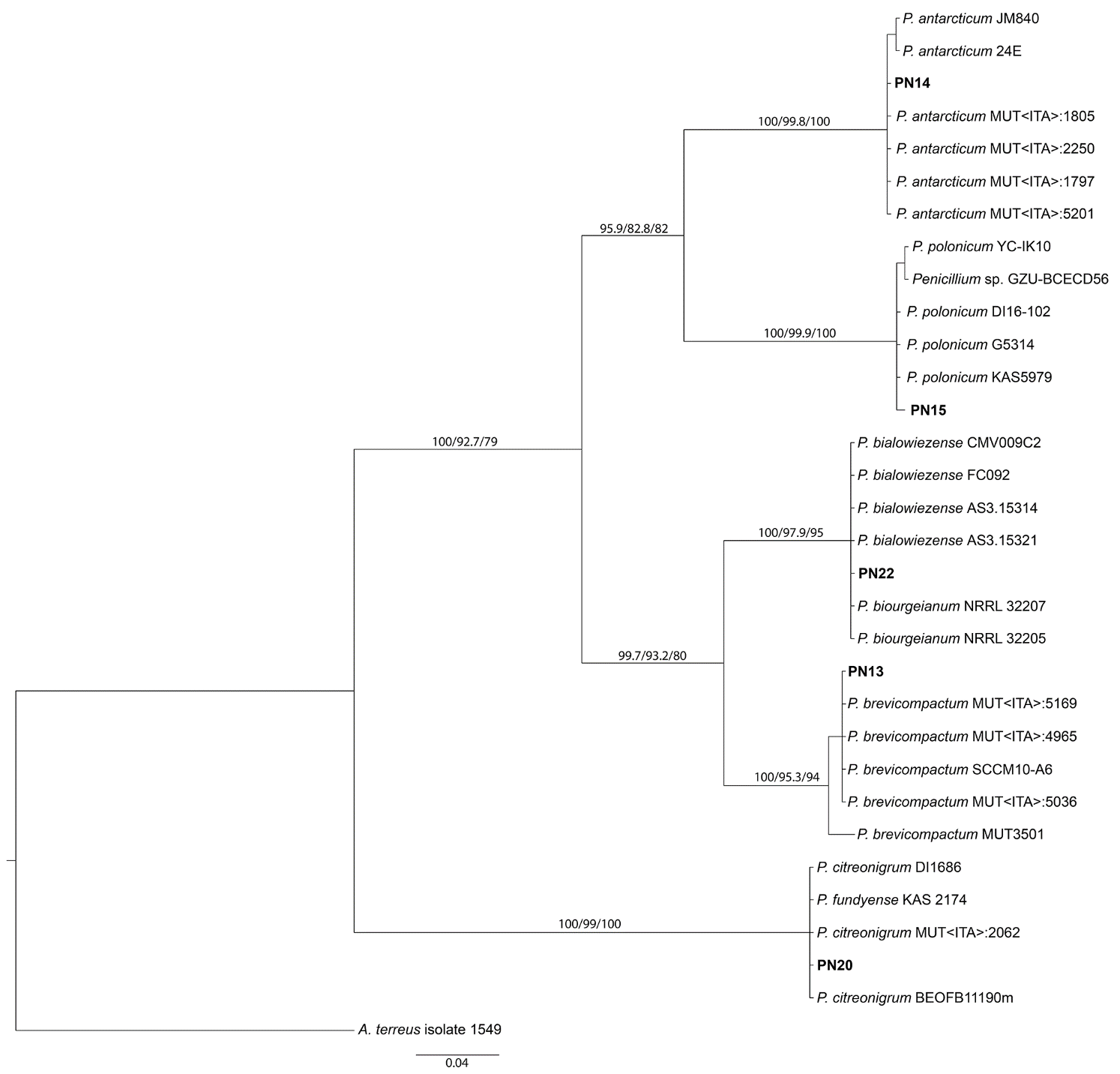


Figure S2. *Penicillium*: Phylogenetic inference based on on β- *tubulin.* Dataset was inferred using the Bayesian method*, Aspergillus terreus* was utilized as outgroup. Branch numbers indicate BYPP (Bayesian posterior probabilities), and sH-aLRT, and BP values from RA-ML analysis. The Bayesian Inference was performed under model as evaluated by jModelTest 2 using Bayesian Information Criterion (TPM1+G, G=0.536).


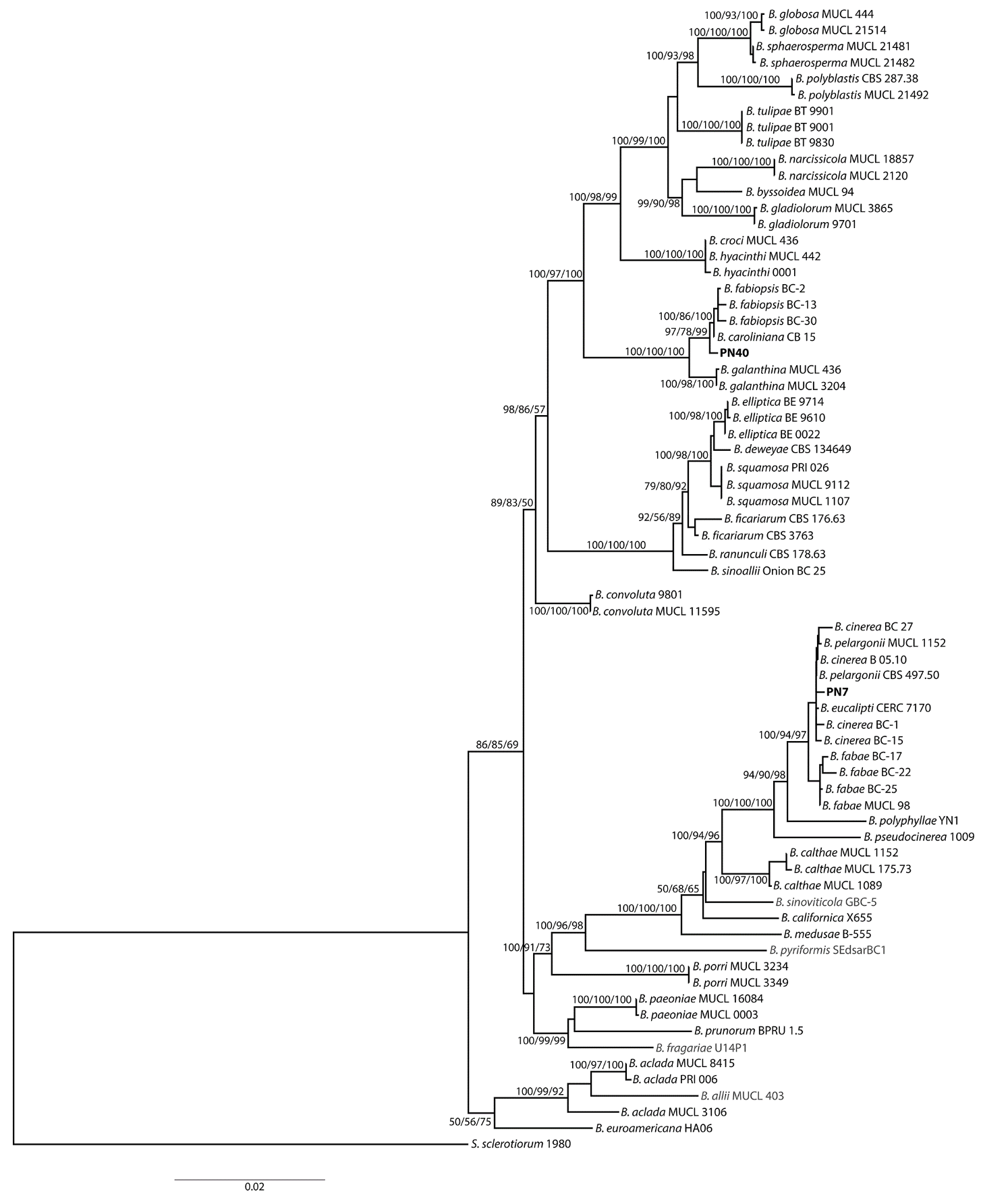


Figure S3. *Botrytis*: Phylogenetic inference based on G3PDH, *rpb*-2, and HSP60, combined dataset was inferred using Maximum Likelihood method (RA-ML)*, Sclerotinia sclerotiorum* was utilized as outgroup. Branch numbers indicate BYPP (Bayesian posterior probabilities), and sH-aLRTand BP values from RA-ML analysis. The Maximum Likelihood inference was performed under different models for each partition as evaluated by ModelFinder (IQ-TREE web server) TNe+I+G4 for partition 1 and 2, and TIM2e+I+G4 for partition 3.
