# Supplementary figures and images for: From marine neglected substrata new fungal taxa of potential biotechnological interest: the case of Pelagia noctiluca
Source: Front Microbiol. 2024 Oct 11;15:1473269. doi: 10.3389/fmicb.2024.1473269 (PMC11502404; doi:10.3389/fmicb.2024.1473269)

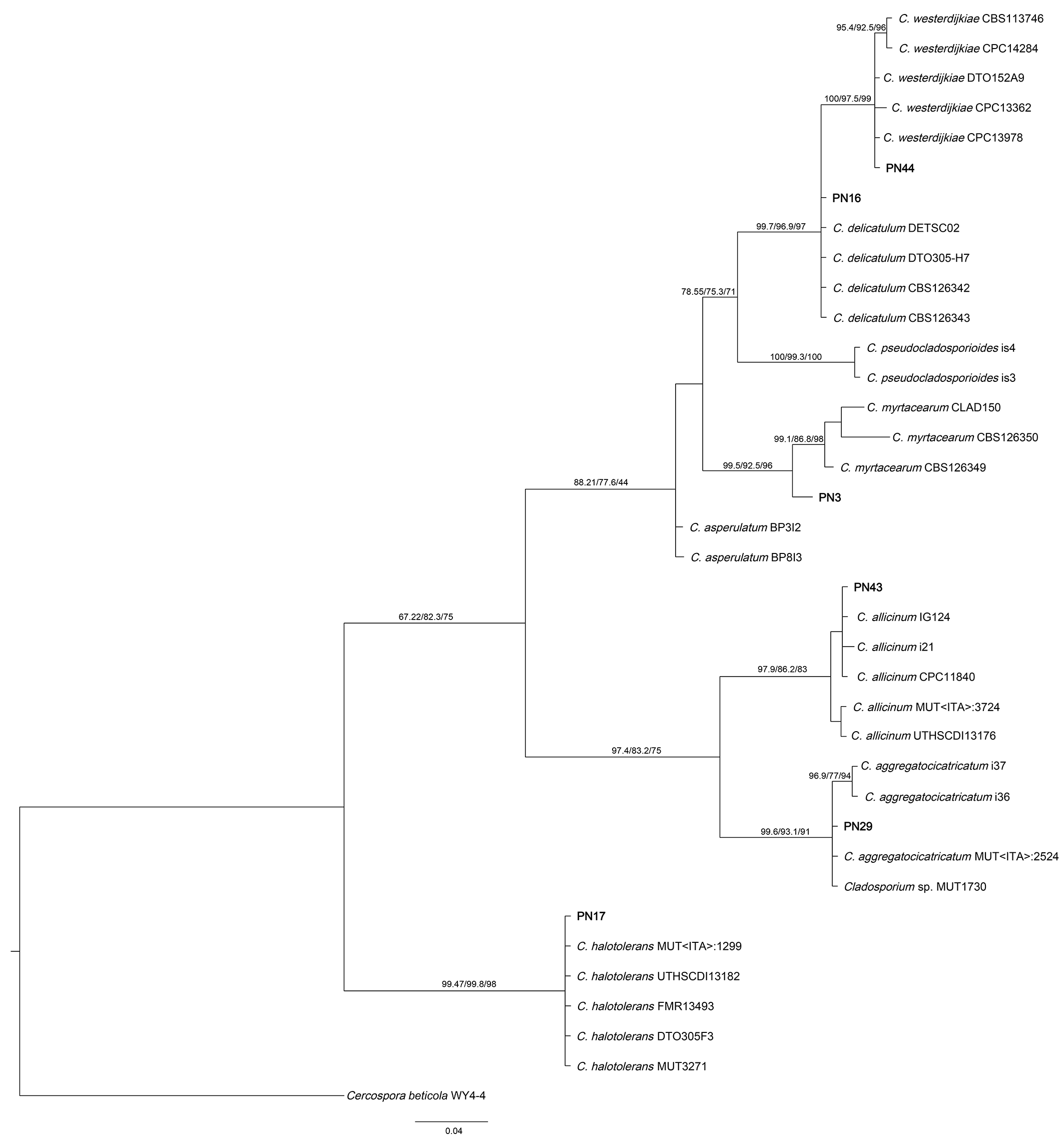

Supplement: Supplementary file 1 [file Data_Sheet_1.ZIP › Supplementary Materials1/Figure S1_Cladosporium.tif]

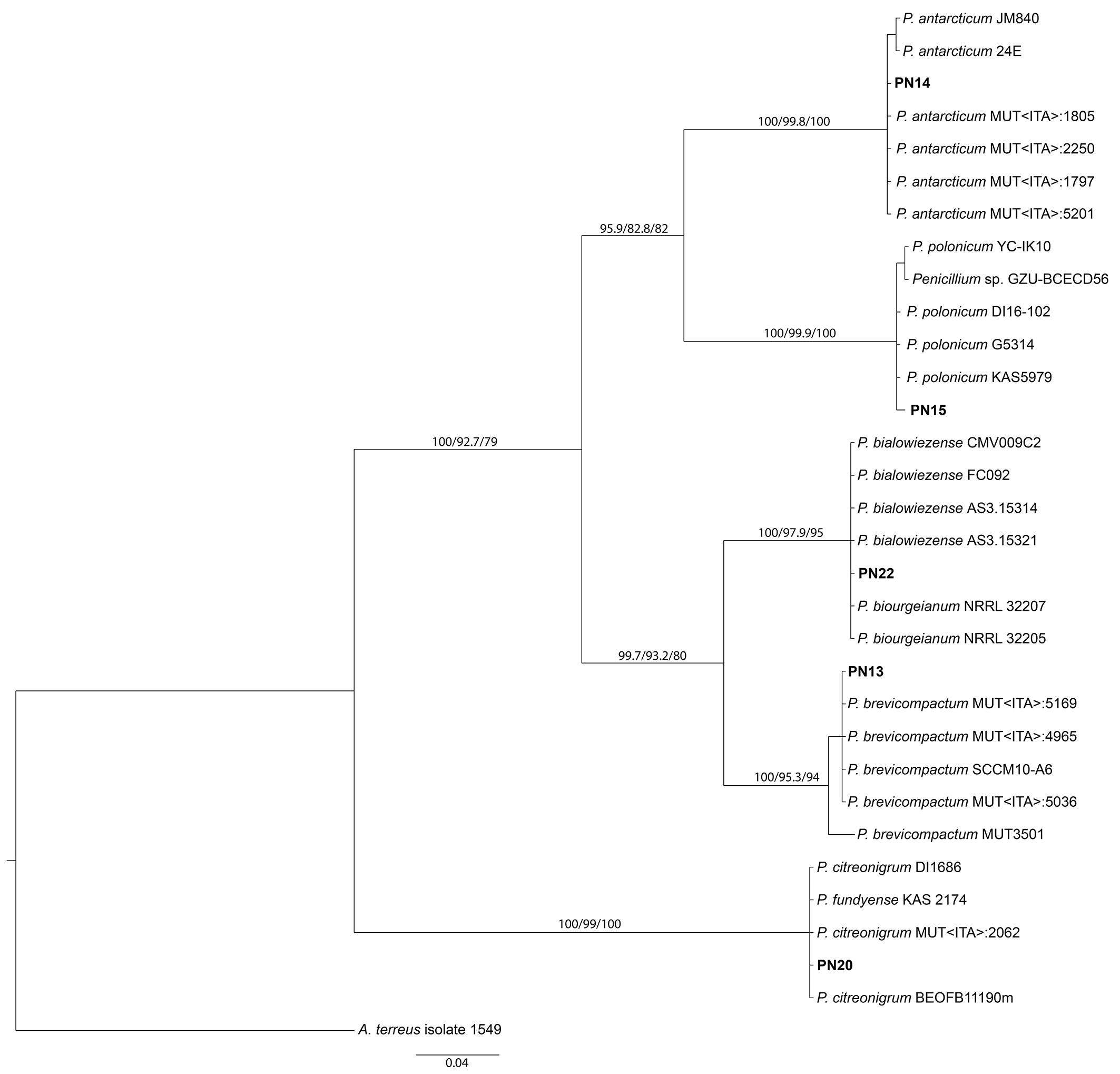

Supplement: Supplementary file 1 [file Data_Sheet_1.ZIP › Supplementary Materials1/Figure S2_Penicillium.tif]

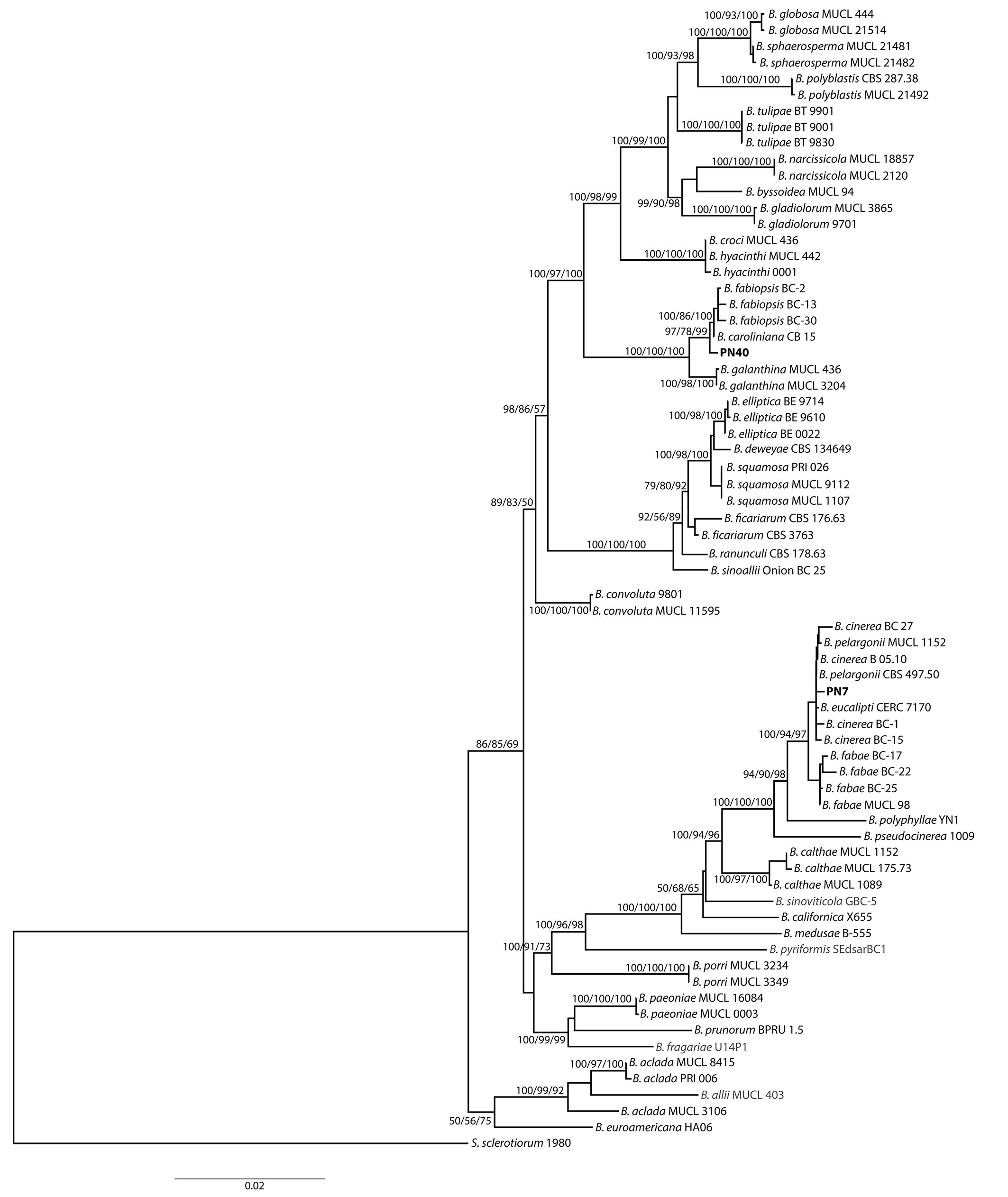

Supplement: Supplementary file 1 [file Data_Sheet_1.ZIP › Supplementary Materials1/Figure S3_Botrytis.tif]
